# NIR-II emissive AIEgen photosensitizers enable ultrasensitive imaging-guided surgery and phototherapy to fully inhibit orthotopic hepatic tumors

**DOI:** 10.1186/s12951-021-01168-w

**Published:** 2021-12-13

**Authors:** Ruizhen Jia, Han Xu, Chenlu Wang, Lichao Su, Jinpeng Jing, Shuyu Xu, Yu Zhou, Wenjing Sun, Jibin Song, Xiaoyuan Chen, Hongmin Chen

**Affiliations:** 1 State Key Laboratory of Molecular Vaccinology and Molecular Diagnostics & Center for Molecular Imaging and Translational Medicine, School of Public Health, Xiamen University, Xiamen, 361102 China; 2 MOE Key Laboratory for Analytical Science of Food Safety and Biology, College of Chemistry, Fuzhou University, Fuzhou, 350108 China; 3 State Key Laboratory of Physical Chemistry of Solid Surfaces, College of Chemistry and Chemical Engineering, Xiamen University, Xiamen, 361005 China; 4 Departments of Diagnostic Radiology and Surgery, Clinical Imaging Research Centre, Centre for Translational Medicine, Nanomedicine Translational Research Program, NUS Center for Nanomedicine, Yong Loo Lin School of Medicine, Singapore, Singapore; 5 Departments of Chemical and Biomolecular Engineering, and Biomedical Engineering, Faculty of Engineering, National University of Singapore, Singapore, Singapore

**Keywords:** NIR-II imaging, AIE, Imaging-guided surgery, Phototherapy, Orthotopic hepatic tumors

## Abstract

Accurate diagnosis and effective treatment of primary liver tumors are of great significance, and optical imaging has been widely employed in clinical imaging-guided surgery for liver tumors. The second near-infrared window (NIR-II) emissive AIEgen photosensitizers have attracted a lot of attention with higher-resolution bioimaging and deeper penetration. NIR-II aggregation-induced emission-based luminogen (AIEgen) photosensitizers have better phototherapeutic effects and accuracy of the image-guided surgery/phototherapy. Herein, an NIR-II AIEgen phototheranostic dot was proposed for NIR-II imaging-guided resection surgery and phototherapy for orthotopic hepatic tumors. Compared with indocyanine green (ICG), the AIEgen dots showed bright and sharp NIR-II emission at 1250 nm, which extended to 1600 nm with high photostability. Moreover, the AIEgen dots efficiently generated reactive oxygen species (ROS) for photodynamic therapy. Investigations of orthotopic liver tumors in vitro and in vivo demonstrated that AIEgen dots could be employed both for imaging-guided tumor surgery of early-stage tumors and for ‘downstaging’ intention to reduce the size. Moreover, the therapeutic strategy induced complete inhibition of orthotopic tumors without recurrence and with few side effects.

**Supplementary Information:**

The online version contains supplementary material available at 10.1186/s12951-021-01168-w.

## Introduction

Liver cancer is globally the seventh most frequent cancer and the third leading cause of cancer-related death [1]. Hepatocellular carcinoma (HCC) is the most common type of primary liver cancer [2]. Surgical resection is one of the highly effective approaches for liver cancer [3]. Clear visualization of the tumor margin during surgery is of utmost importance for the real-time surgical guidance [4, 5].

As a sensitive, noninvasive, and radiation-free technology, the first near-infrared window (NIR-I) fluorescence imaging has been widely employed in clinical imaging-guided surgery for liver tumors and retinal angiography [6, 7]. However, the following disadvantages limit further applications of NIR dyes: (1) autofluorescence of tissues, which decreases the sensitivity and signal-to-noise ratio; (2) photobleaching; (3) aggregation-caused quenching (ACQ) effect. Therefore, much effort has been made to develop new luminescent agents to achieve excellent imaging ability [8–13]. In 2001, Tang group found an optical property of aggregation-induced emission (AIE) based on the restriction of intramolecular motion mechanism [14]. Moreover, AIE could be extended to the second near-infrared window (NIR-II, 900–1700 nm) [15–20]. NIR-II allows for higher-resolution bioimaging with deeper penetration (*ca.* 5–20 mm) compared with the visible and the NIR-I bands (*ca.* 1–3 mm) [21–23]. These advantages make NIR-II fluorescent agents suitable for broad applications in whole-body angiography, organ visualization, and tumor diagnosis and imaging-guided therapy [24–28].

Metal-containing inorganic NIR-II dots have been shown to have higher luminescence than the NIR-I equivalents; however, one major concern is their potential toxicity after decomposition within the body [29–31]. As an alternative, NIR-II organic dyes have attracted more attention because of their relatively low toxicity, good biocompatibility and pharmacokinetics, as well as their well-defined structure [32–35]. Although many NIR-II fluorophores have been explored as imaging agents, the emission is easily quenched due to the dominant nonradiative decay caused by intense intermolecular π–π interactions [35–40]. Furthermore, most NIR-II fluorescent dyes have a single imaging function and need to be combined with NIR-II activated photosensitizers to realize phototherapy [41–47]. Recent studies have shown that NIR-II agents could achieve both fluorescence and photothermal (PTT) or photodynamic (PDT) processes, thereby boosting NIR-II imaging-guided surgery to achieve optimal effect [48–56]. Surgical resection is the treatment option for a small number (< 30%) of patients with early-stage liver cancers who have normal liver function [57]. Given that patients with large or multiple HCC cannot undergo surgery, ‘downstaging’ pretreatment may be required to reduce the size or number of active tumors [57–60]. Therefore, it is urgent to design and synthesize NIR-II emissive agents with both fluorescence imaging and phototherapeutic ability in their aggregation state.

**Scheme 1:**
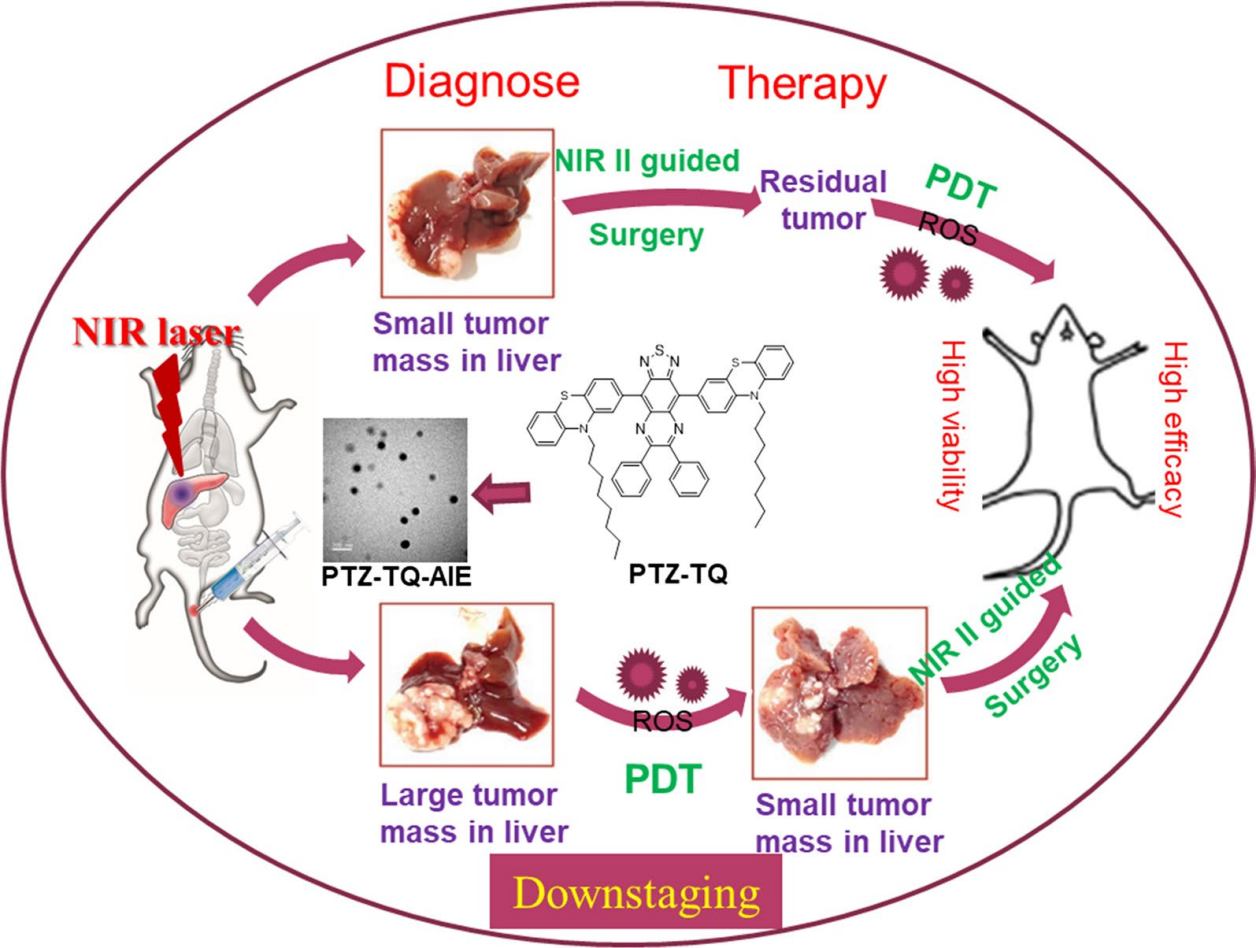
Schematic illustration of NIR-II emissive AIEgen photosensitizer PTZ-TQ that enables ultrasensitive imaging-guided surgery and phototherapy to fully inhibit orthotopic hepatic tumors

Herein, we designed and successfully synthesized a novel AIE-based NIR-II photosensitizer with donor–acceptor–donor (D–A–D) structure (Scheme 1). We compared the optical properties of the photosensitizer with clinically used indocyanine green (ICG) and evaluated its ability to generate reactive oxygen species (ROS). Furthermore, we investigated the efficacy of imaging-guided surgical resection of orthotopic early-stage liver tumor and ‘downstaging’ intention of large HCC.

## Results and discussion

### Synthesis and characterization of PTZ-TQ dye

**Fig. 1:**
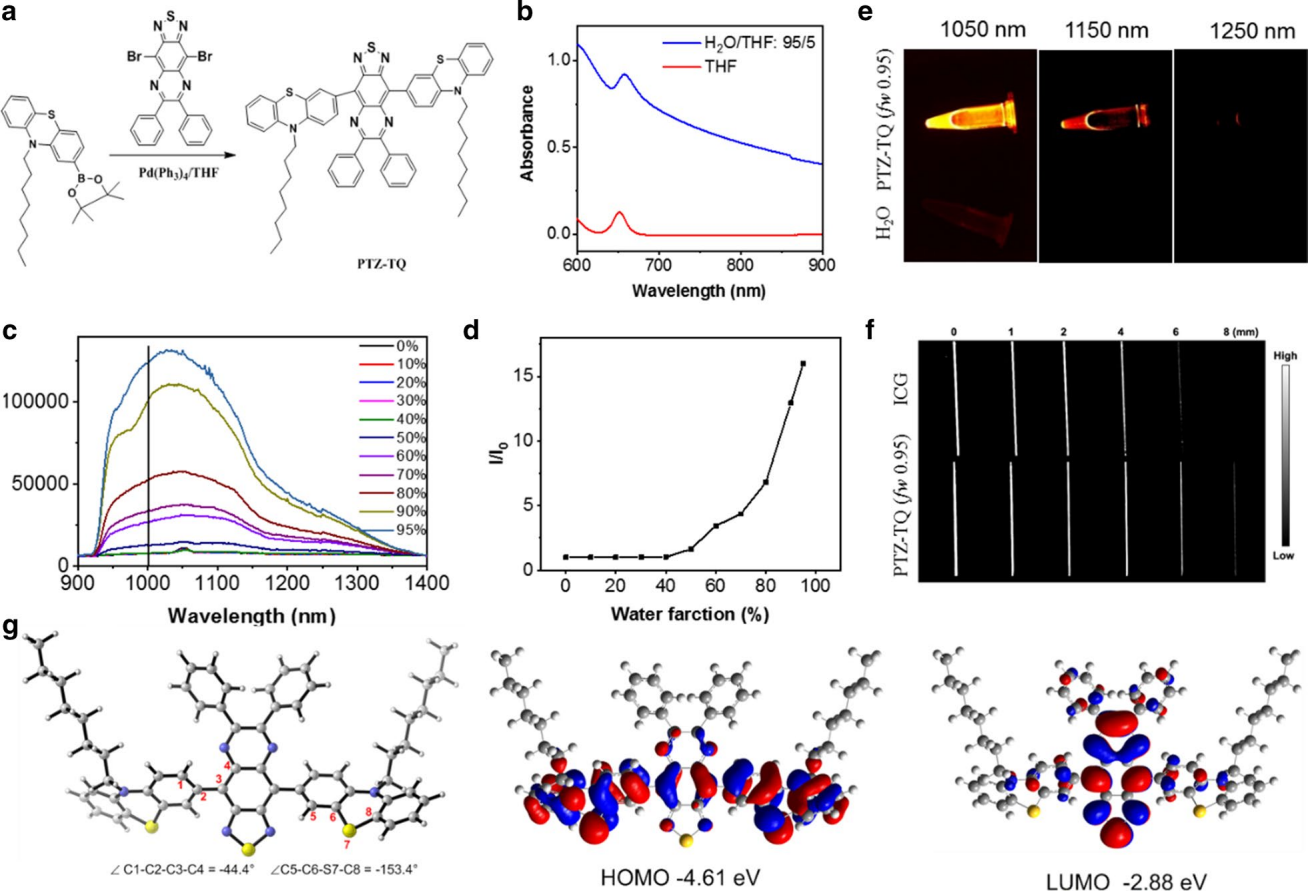
**a** Synthetic route of PTZ-TQ molecule. **b** Absorption spectra of PTZ-TQ in tetrahydrofuran (THF) and in THF/H_2_O with 95% water volume fraction (*f*_*w*_). **c** Emission of PTZ-TQ in THF/H_2_O (*f*_*w*_, 0% to 95%) under 808-nm excitation. **d** Emission intensity ratio (*I*/*I*_0_, calculated from (**c**) of PTZ-TQ under different *f*_*w*_ (*I*_0_ is the emission intensity of PTZ-TQ in pure THF). **e** NIR-II fluorescence images of PTZ-TQ in THF/H_2_O (*f*_*w*_, 95%) and pure THF (50 μg/mL, 808-nm excitation, 1050, 1150, 1250 band-pass filter). **f** Signal intensity of PTZ-TQ and ICG through 1% intralipid solution (excitation 808 nm, 1050-nm band-pass filter). **g** Calculated HOMOs and LOMOs of PTZ-TQ [utilizing B3LYP functional and 6–31 g (**d**) basis set]

A Suzuki cross-coupling reaction between compound **3** and compound **6** formed the final dye with a D–A–D architecture, 7,7ʹ-(6,7-diphenyl-[1,2,5]thiadiazolo[3,4-g]quinoxaline-4,9-diyl)bis(10-octyl-10H-phenothiazine) (termed PTZ-TQ) (Fig. 1a). The total synthesis route is shown in Additional file 1: Fig. S1. The D–A–D structure was confirmed by nuclear magnetic resonance (NMR) spectroscopy and ESI–MS analysis (Additional file 1: Figs. S2–S12). The D–A–D structure exhibited excellent NIR absorption property at 650 nm in THF and showed enhanced absorption at 660 nm, with the absorption tail extending to 900 nm in THF–water mixtures with water volume fractions (*f*_*w*_) of 95% (Fig. 1b). The photoluminescence (PL) spectra of PTZ-TQ in THF–H_2_O were measured in 0–95% *f*_*w*_. As indicated, under 808-nm excitation, the PTZ-TQ solution emitted almost no luminescence even after increasing the *f*_*w*_ up to 40%. Then, the emission of PTZ-TQ enhanced dramatically when the *f*_*w*_ exceeded 50% (Fig. 1c). Together with the plot of emission intensity at 1000 nm vs. *f*_*w*_, the higher *f*_*w*_ and the stronger emission indicate typical AIE characteristics (Fig. 1d) [14].

The emission of PTZ-TQ was measured in the THF–water mixture with an *f*_*w*_ of 95%. Under 808-nm excitation, the PTZ-TQ solution exhibited significant NIR-II fluorescence signals at 1050 nm and 1150 nm (Fig. 1e). Then, the penetration depth of fluorescence at 1050 nm was studied using 1% intralipid as the mimic of tissue. Compared with commercial ICG dye, edges were clearly visualized up to a depth of 6 mm in PTZ-TQ (Fig. 1f). Density functional theory (DFT) calculations were carried out to explore the relationship between the structure and the emission property of the PTZ-TQ. Obviously, the highest occupied molecular orbitals (HOMOs) were delocalized along the whole backbone, while the lowest unoccupied molecular orbitals (LUMOs) were mainly distributed on electron acceptor moieties, indicating the intramolecular charge transfer of the fluorophore [61]. It is generally believed that stronger D–A effect is associated with smaller singlet–triplet energy gap (Fig. 1g). The smaller energy band gap (1.73 eV) would endow PTZ-TQ with longer absorption and would greatly promote the generation of ROS [62, 63]. These features are remarkably beneficial for PTZ-TQ to achieve NIR-II AIE imaging and therapy.

### Preparation and characterization of PTZ-TQ-AIE dots

**Fig. 2:**
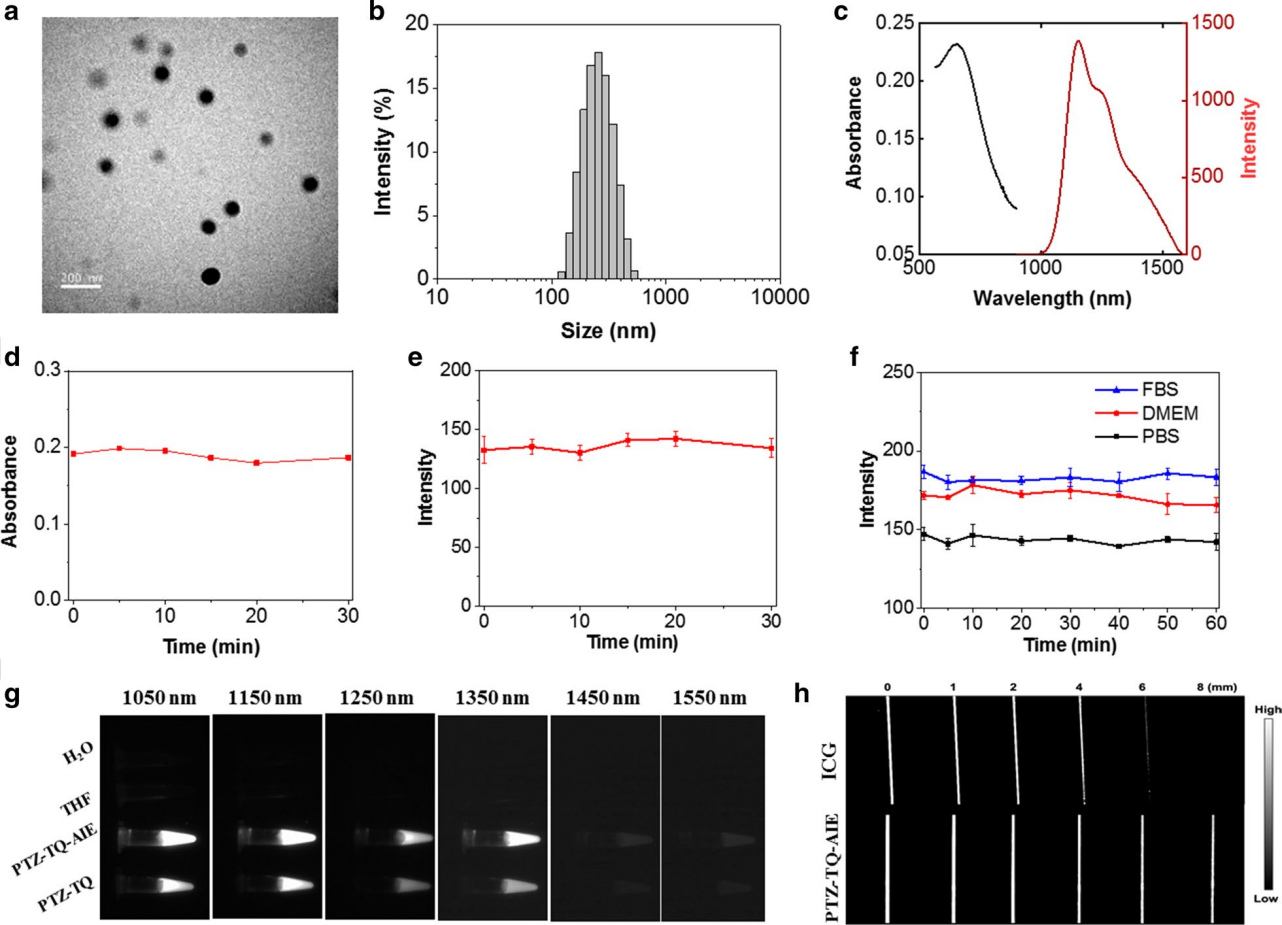
**a** TEM image of PTZ-TQ-AIE dots. **b** Size distribution of the PTZ-TQ-AIE dots in water. **c** UV–vis-NIR absorption spectrum and NIR-II fluorescence emission spectrum of the PTZ-TQ-AIE dots in aqueous solution (808-nm excitation). **d** Photostability of the PTZ-TQ-AIE dots in PBS, FBS, and DMEM under intermittent 808-nm laser (0.25 W/cm^2^) irradiation for 60 min. **e** Absorbance and **f** NIR-II fluorescence emission under continuous 808-nm laser (0.25 W/cm^2^) irradiation for 30 min. **g** NIR-II fluorescence images of PTZ-TQ-AIE dots in water (808-nm excitation, 1050, 1150, 1250, 1350, 1450, and 1550 nm band-pass filters). **h** Signal intensity of PTZ-TQ-AIE dots and ICG through various thicknesses of 1% intralipid solution (excitation: 808 nm, 1050-nm band-pass filter)

PTZ-TQ-AIE dots were prepared by a nanoprecipitation method using 1,2-distearoyl-sn-glycero-3-phosphoethanolamine-*N*-[amino(polyethylene glycol)-3400] (DSPE-PEG_3400_-NH_2_) as the encapsulation matrix. The size of the PTZ-TQ-AIE dots was about 80 nm as measured by transmission electron microscopy (TEM) and 255 nm as measured by dynamic light scattering (DLS) analysis (Fig. 2a, b). After loading into DSPE-mPEG_3400_-NH_2_, PTZ-TQ-AIE dots showed a positive surface charge (about 7 mV) and had high stability in buffer and biological fluids (PBS, DMEM, and FBS solution) (Additional file 1: Figs. S13–S16). The UV–vis-NIR absorption and NIR-II fluorescence emission spectra of the PTZ-TQ-AIE dots in water showed that the absorption peak at 675 nm was extended to 900 nm, and the fluorescence emission peak located at 1150 nm with emission tail was extended to close to 1600 nm (Fig. 2c). The quantum yield (*QY*) of PTZ-TQ-AIE dots in aqueous solution was 0.3% under 808-nm laser excitation, using dye 4-(7-(2-phenyl4H-1-benzothiopyran-4-ylidene)-4-chloro-3,5-trimethylene1,3,5-heptatrienyl)-2-phenyl-1-benzothiopyrylium perchlorate (IR26) in dichloroethane (DCE) (*QY* = 0.5%) as the reference (Additional file 1: Fig. S17) [64]. Importantly, the PTZ-TQ-AIE dots showed superior photostability in media (PBS, FBS and DMEM). No obvious changes were observed in the absorption and emission spectra of the PTZ-TQ-AIE dots when the mixture was irradiated continuously with a 808-nm laser at a power density of 0.25 W/cm^2^ for up to 30 min (Fig. 2d, e). Furthermore, the luminescence intensities of PTZ-TQ-AIE dots in phosphate-buffered saline (PBS), Dulbecco’s modified Eagle medium (DMEM), and fetal bovine serum (FBS) showed no changes after continuous irradiation for 60 min under the same density (Fig. 2f). The photostability was far better than that of the commercial ICG dye, indicating that PTZ-TQ-AIE dots have an excellent potential for long-term in vivo fluorescent imaging.

Figure 2c shows that PTZ-TQ-AIE dots emitted luminescence in the NIR-IIb region (1400–1700 nm); so, we investigated the fluorescence properties of PTZ-TQ-AIE dots in NIR-IIb. The NIR-IIb fluorescence intensity was measured by different band-pass filters. PTZ-TQ-AIE dots exhibited better NIR-IIa (1000–1300 nm) and NIR-IIb (1400–1700 nm) fluorescence signals than PTZ-TQ (*f*_*w*_ = 95%) (Fig. 2g). Furthermore, the penetration depths of fluorescence at 1050 nm indicated that PTZ-TQ-AIE dots resolved sharper edges of the capillary at a depth of up to 8 mm; under the same conditions, ICG showed similar resolution only at a depth of 4 mm (Fig. 2h).

**Fig. 3:**
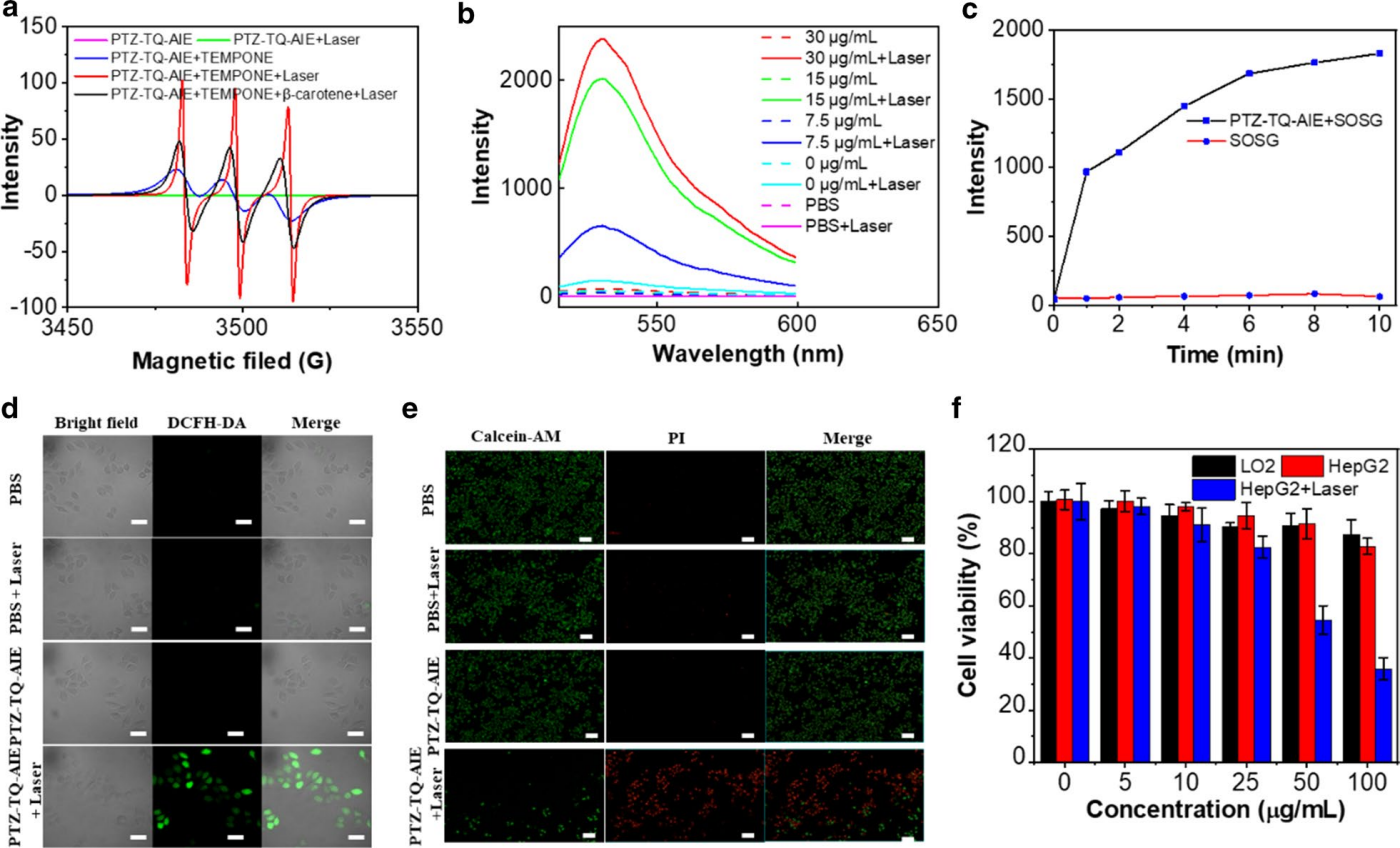
**a** Electron paramagnetic resonance (EPR) spectra to detect ^1^O_2_ generation. **b** Fluorescence spectra of ^1^O_2_ probes with various concentrations of PTZ-TQ-AIE dots from 0 to 30 μg/mL were recorded (solid line: with laser, dotted line: without laser). **c** Fluorescence intensities of ^1^O_2_ probes with PTZ-TQ-AIE dots under irradiation for different time durations (808-nm NIR laser, 0.25 W/cm^2^). **d** Intracellular ROS production by DCFH-DA in HepG2 cells (50 μg/mL; 808 nm, 0.25 W/cm^2^, 10 min; scale bar: 200 μm). **e** Fluorescence images of HepG2 cells co-stained by Calcein-AM (green fluorescence for live cells) and PI (red fluorescence for dead cells) (50 μg/mL; 808 nm, 0.25 W/cm^2^, 10 min; scale bar: 200 μm). **f** In vitro cytotoxicity of PTZ-TQ-AIE dots against LO2 cells, HepG2 cells with and without 808-nm NIR laser (0.25 W/cm^2^) after 24-h incubation

The ROS generation ability of the PTZ-TQ-AIE dots was assessed using dichlorodi-hydrofluorescein diacetate (DCFH-DA) as the indicator. Fluorescence of DCFH-DA increased sharply with increasing irradiation time in the presence of PTZ-TQ-AIE dots (808 nm, 0.25 W/cm^2^) (Additional file 1: Fig. S18). For further verification, electron paramagnetic resonance (EPR) was applied to verify the generation of ROS. After incubating 5 mM of an ROS indicator, 4-oxo-2,2,6,6-tetramethylpiperidinooxy (TEMPONE), with PTZ-TQ-AIE dots in solution, followed by irradiation for 5 min for EPR measurements, the EPR signal decreased when β-carotene was added (Fig. 3a), demonstrating the efficient production of ^1^O_2_. To understand the generated species, singlet oxygen sensor green (SOSG) was firstly used to assess the ^1^O_2_ generation. Under the irradiation (808 nm, 0.25 W/cm^2^) in the presence of PTZ-TQ-AIE dots, the fluorescence of SOSG increased sharply with the increase in irradiation time, confirming that ^1^O_2_ was the predominant ROS (Fig. 3b, c). Subsequently, the ^1^O_2_ quantum yield of the PTZ-TQ-AIE dots was calculated as 10% using ICG as a reference (12%) (Additional file 1: Fig. S19).

As PTZ-TQ-AIE dots have excellent NIR-II emission and ROS generation ability, the potential of such AIE dots in cancer diagnostics and therapeutics was investigated. As shown in Fig. 3d, under irradiation (808 nm, 0.25 W/cm^2^), bright green fluorescence of DCFH-DA was displayed in the group of PTZ-TQ-AIE dots plus laser, indicating that ROS generation was efficiently induced, and the intracellular ROS generation was quantified by flow cytometry (Additional file 1: Fig. S20). The efficient generation of ROS resulted in cell death (Fig. 3e). However, only irradiation or PTZ-TQ-AIE dots showed negligible red fluorescence, implying the good biocompatibility of the PTZ-TQ-AIE dots and limited harm of the laser irradiation alone (Fig. 3e). Using hepatic LO2 normal cells as a control, in vitro cytotoxicity studies showed that PTZ-TQ-AIE dots had almost no obvious toxic effects on both normal cells and cancer cells at concentrations as high as 100 μg/mL (Fig. 3f). Additionally, PTZ-TQ-AIE dots and irradiation resulted in a significant decrease in cell viabilities, demonstrating in vitro photodynamic therapy (PDT) efficacy with minor side effects of the PTZ-TQ-AIE dots themselves.

### In vivo imaging and therapy

**Fig. 4:**
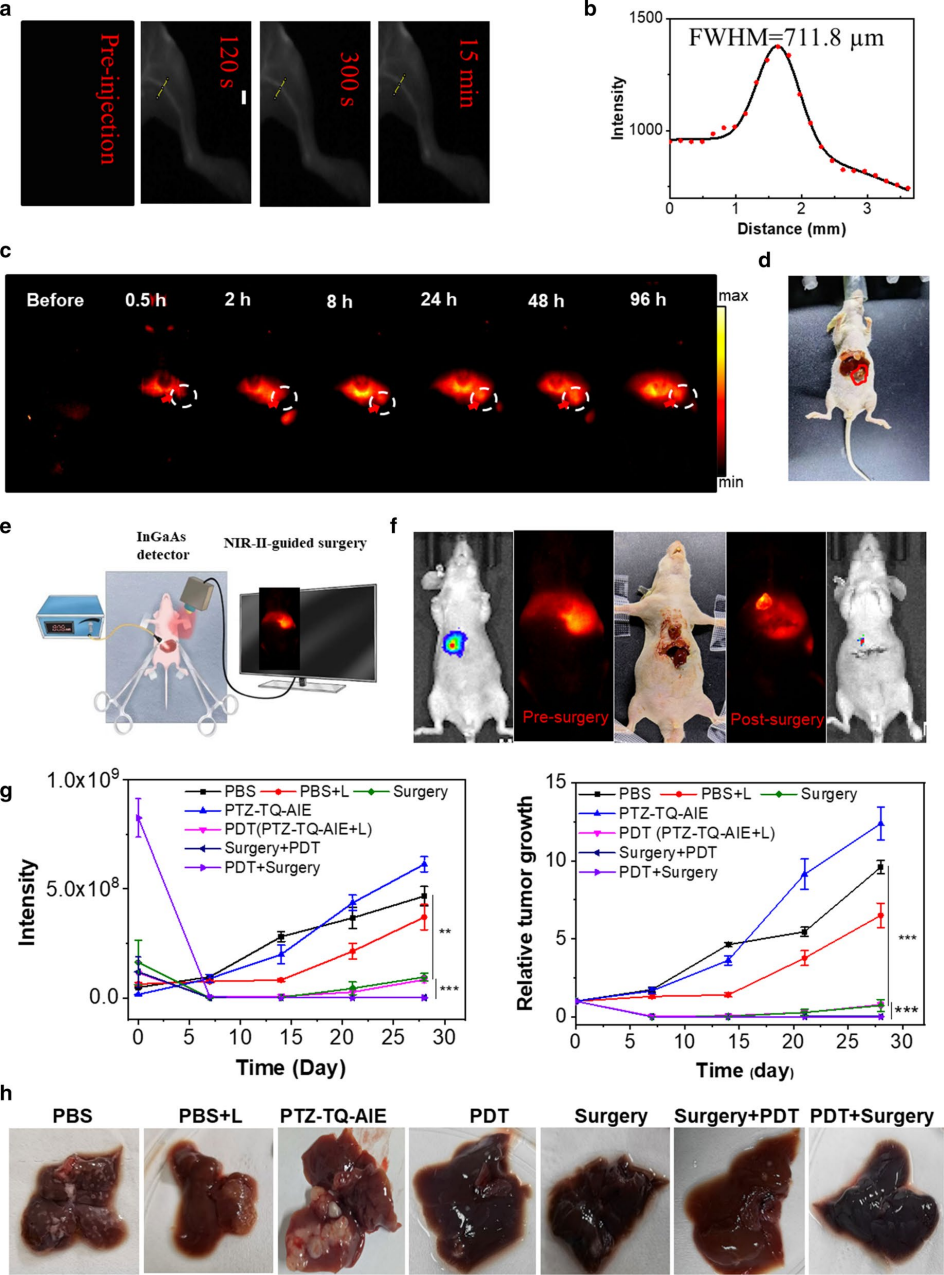
**a** Intravital long-term hindlimb vasculature NIR-II imaging (1250-nm band-pass filter, 500 ms, 808-nm excitation) at 120 s, 300 s, and 15 min after tail vein injection of the PTZ-TQ-AIE dots. **b** The vessel FWHM width based on the cross-sectional intensity profile measured along the yellow line in (**a**) (120 s) with the peak fitted to Gaussian functions (the black curve is the Gaussian fit to the profile). **c** Representative in vivo long-term NIR-II fluorescence images (808-nm excitation, 1250-nm band-pass filter, 500 ms, n = 3) of the orthotopic liver cancer at different time points after tail vein injection of the PTZ-TQ-AIE dots (0.2 mL, 0.5 mg/mL). The white circles indicate the tumor. **d** The pictures of liver and tumor. Scale bar: 200 μm. **e** Illustration of the NIR-II optical imaging-guided tumor resection. **f** The NIR-II and bioluminescence imaging in surgery. **g** The relative tumor growth curve and corresponding fluorescence intensity of the tumor-bearing mice. Data are shown as means (n = 3). **h** Photographs of dissected liver after treatments

To further investigate the capabilities of NIR-II imaging and photodynamic therapy, the biocompatibility and biodistribution of PTZ-TQ-AIE dots were first evaluated in vivo. After intravenous injection of PTZ-TQ-AIE dots into healthy BALB/c mice (200 μL, 500 μg/mL), blood samples of the mice were collected, and serum chemistry and blood cells were analyzed. No significant changes in the main blood cell counts (Additional file 1: Fig. S21) were seen at day 1 and day 7 post-injection (p.i.), suggesting high biocompatibility of PTZ-TQ-AIE dots. Furthermore, a biodistribution study of PTZ-TQ-AIE dots in vitro was also carried out to evaluate their distribution in major organs at 168 h after injection (Additional file 1: Fig. S22). High accumulation was found in the liver and spleen, indicating that the clearance pathway of PTZ-TQ-AIE dots was through the hepatobiliary system. Compared with the commercial ICG, PTZ-TQ-AIE dots exhibited a relatively long blood-circulation half-life of 61 ± 21 min (Additional file 1: Fig. S23) [65, 66]. After confirming its good biocompatibility, the capability of vasculature imaging using the PTZ-TQ-AIE dots was then investigated. The vasculature was clearly visualized through NIR-II imaging after 2, 5, and 15 min post-injection (Fig. 4a). Furthermore, the resolution (711.8 μm) of the hind limb vasculature was reached via measuring the Gaussian-fitted full width at half maximum (FWHM) (Fig. 4b), which would be adequate for surgical operation [6]. To evaluate the imaging and therapy for deep tissues, an orthotopic liver tumor model was established in nude mice in line with our previous procedure [67]. Human hepatocellular carcinoma HepG2 cells were transfected with firefly luciferase and then inoculated into the right liver lobe by laparotomy. At day 10 after the operation, bioluminescence imaging (BLI) was carried out to monitor the tumor growth. After confirming the successful establishment of the orthotopic liver tumor, the PTZ-TQ-AIE dots (200 μL, 500 μg/mL) were intravenously injected into tumor-bearing mice. As observed, NIR-II fluorescence in both normal liver and the tumor sites was continuously recorded under the excitation of an 808-nm laser. At 2 h post-injection, ex vivo results indicated NIR-II fluorescence signals at the tumor site, which were lower than those at normal liver tissues; importantly, the lower NIR-II signals in the tumors clearly identified the boundary between the tumor and normal liver organs (Additional file 1: Fig. S24). Notably, the accumulation of PTZ-TQ-AIE dots in the tumors reached the maximum at 48 h post-injection (Fig. 4c, Additional file 1: Fig. S25), and bright and sharp NIR-II signals were clearly visualized in the tumor regions at 48 h post-injection. The signal to noise ratio (SNR) gradually increased with time and reached the maxima at 48 h for NIR-II fluorescence imaging (Additional file 1: Fig. S26). To further confirm the tumor position, the whole liver tissue was photographed at 96 h post-injection (Fig. 4d); the location was in accordance with that observed on NIR-II images in vivo (circles in Fig. 4c). These results demonstrate that the boundary between the tumor and normal liver organs can be clearly identified at 2 h post-injection by negative enhancement or at 48 h post-injection by positive enhancement. So, the PTZ-TQ-AIE dots can be employed as imaging-guided surgery both at 2 h and 48 h post-injection, which can provide personalized treatment choice according to the size of tumors.

### NIR-II optical imaging surgical guidance

Delineating the tumor margin is the key step for precise tumor resection and is essential for tumor curation [4, 68, 69]. As indicated, the tumor margins were clearly delineated, and surgical resection of the tumors was then performed at 2 h or 48 h post-injection of PTZ-TQ-AIE dots using the NIR-II imaging system (Additional file 2: Videos S1 and S2). The resected tissues showed that most of the tumor was removed from the liver, which was confirmed by the bright NIR-II fluorescence signals from the isolated tumor and the hematoxylin and eosin (H&E) results. The boundary between the tumor and normal tissue was clearly visualized, confirming the successful tumor resection under the guidance of NIR-II optical imaging (Figs. 4e, f, Additional file 1: Fig. S27).

### Surgery/PDT on the orthotopic hepatic tumor

In clinical setting, the tumor removal operation sometimes cannot be performed because of the presence of multiple smaller tumors or a very large tumor [70, 71]. Multiple treatments, a common methodology in the clinical setting, is a good choice for patients with large or multiple HCC [72]. Given that PTZ-TQ-AIE dots are strong ROS generators, we investigated the feasibility of PTZ-TQ-AIE dots in photodynamic therapy to reduce the tumor size for further surgical resection and prevent the recurrence of tumors.

Thus, we applied different therapy treatments: for large tumors, we first applied PDT treatments to reduce the tumor size, followed by surgery to minimize the injury of the liver in the therapeutic process (named as PDT + Surgery); for small tumors, we first applied surgery and then PDT treatment for the residual tumors (named as Surgery + PDT). The therapeutic processes were monitored by BLI (Additional file 1: Fig. S28a). The tumor growth with various treatments was summarized using the total BLI intensity and relative tumor growth. As indicated, the BLI signals in control groups (PBS, PBS + Laser, and PTZ-TQ-AIE dots) increased rapidly (Fig. 4g), indicating a high tumor growth rate. Importantly, single therapeutic process (PDT or surgery) induced significant decrease of BLI signals, demonstrating the efficacy of single imaging-guided surgery or PDT in tumor management. However, the tumors in both single therapeutic processes relapsed over time (green and pink curves in Fig. 4g). The therapy in the early stage achieved therapeutic efficacies similar to that of the single therapeutic process but without tumor recurrence during the observation period (purple and dark blue curves in Fig. 4g).

### ‘Downstaging’ intention for orthotopic hepatic tumors

Notably, we simulated a large HCC model that was not suitable for surgery [73–75] (purple curve in Fig. 4g). In the clinical setting, ‘downstaging’ intention involves pretreatment to reduce the size or number of active tumors. In our protocol, we first employed PDT to reduce the size. After PDT treatment, the BLI signals decreased sharply at day 7 post-treatment, and then, surgery was conducted to achieve almost complete tumor inhibition (purple curve in Fig. 4g). Magnetic resonance imaging (MRI) of the livers in vivo and photographs ex vivo confirmed that there were almost no tumor nodules in the multiple treatments groups (Fig. 4h, Additional file 1: Fig. S29). Moreover, PTZ-TQ-AIE dots showed good biocompatibility, as indicated by the assessment of body weight, histological analysis of organs, and blood analysis (Additional file 1: Figs. S28b, S30).

## Conclusions

In summary, biocompatible NIR-II emissive AIEgen photosensitizers enable ultrasensitive imaging-guided surgery and phototherapy to fully inhibit orthotopic hepatic tumors. Compared with ICG, the PTZ-TQ-AIE dots showed bright and sharp NIR-II emission at 1250 nm, which extended to 1600 nm with high photostability. Moreover, the PTZ-TQ-AIE dots were able to efficiently generate ROS for photodynamic therapy. Investigations of orthotopic liver tumors in vitro and in vivo demonstrated that PTZ-TQ-AIE dots could be employed both for imaging-guided tumor surgery of early-stage tumors and for ‘downstaging’ intention to reduce the tumor size. Moreover, the current therapy achieved full inhibition of orthotopic tumors without recurrence.

## Supplementary Information

**Additional file 1: Figure S1.** Synthetic route of the PTZ-TQ molecule. **Figure S2.** ^1^H NMR spectrum of 3 in CDCl_3_. **Figure S3.** ^13^C NMR spectrum of 3 in CDCl_3_. **Figure S4.** ^1^H NMR spectrum of 4 in DMSO-*d*_*6*_. **Figure S5.** ^13^C NMR spectrum of 4 in DMSO-*d*_*6*_. **Figure S6.** ^1^H NMR spectrum of 5 in DMSO-*d*_*6*_. **Figure S7.** ^13^C NMR spectrum of 5 in DMSO-*d*_*6*_. **Figure S8.** ^1^H NMR spectrum of 6 in DMSO-*d*_*6*_. **Figure S9.** ^13^C NMR spectrum of 6 in DMSO-*d*_*6*_. **Figure S10.** ^1^H NMR spectrum of PTZ-TQ in pyridine-*d*_*5*_. **Figure S11.** ^13^C NMR spectrum of PTZ-TQ in pyridine-*d*_*5*_. **Figure S12.** The mass spectrum of PTZ-TQ. m/z: calcd. 958.39, found: 959.39 for [M]^+^. **Figure S13.** The zeta potentials of PTZ-TQ and PTZ-TQ-AIE dots. **Figure S14.** The stability evaluation of PTZ-TQ-AIE dots in PBS based on hydrodynamic size. PTZ-TQ-AIE dots were incubated in PBS at different time points. The hydrodynamic size at 0 h, 2 h, 4 h, 8 h, 12 h, 24 h, 48 h and 72 h, had no obvious changes compared with 0 h. **Figure S15.** The stability evaluation of PTZ-TQ-AIE dots in DMEM based on hydrodynamic size (a-d). PTZ-TQ-AIE dots were incubated in DMEM at different time points. The hydrodynamic size at 0 h (a), 12 h (b), 24 h (c), 48 h (d) had no obvious changes compared with 0 h. **Figure S16.** The stability evaluation of PTZ-TQ-AIE dots in 5% FBS based on hydrodynamic size (a-d). PTZ-TQ-AIE dots were incubated in 5% FBS at different time points. The hydrodynamic size at 0 h (a), 12 h (b), 24 h (c), 48 h (d) had no obvious changes compared with 0 h. **Figure S17.** Fluorescence quantum yield measurements of PTZ-TQ-AIE dots in water. Absorbance and fluorescence spectra of IR26 in DEM (a-c), and PTZ-TQ-AIE dots in water (d-f). The integrated fluorescence was plotted against absorbance for both IR26 and fluorophores and fitted into a linear function, linear fit of IR26 (c) and PTZ-TQ-AIE dots (f). **Figure S18.** (a) ROS generation of PTZ-TQ-AIE dots with different concentrations. (b) ROS generation of PTZ-TQ-AIE dots with different times. The light source: 808 nm NIR laser (0.25 W/cm2) (unreal thread have no laser and real thread have laser). **Figure S19.** Decrease in absorbance of DPBF at 417 nm in the presence of PTZ-TQ-AIE dots (a) and ICG (b) as a function of irradiation time. **Figure S20.** ROS levels in HepG2 cells analyzed by flow cytometry. **Figure S21.** In vivo blood test including red blood cells, platelet, and white blood cell count of healthy mice injected with saline, PTZ-TQ-AIE dots for 1d and 7d. **Figure S22.** (a) The ex vivo biodistribution analysis of PTZ-TQ-AIE dots in BABL/c normal mice at 168 h under an 808 nm laser excitation (1250 nm bandpass filter, 300 ms). (b) The ex vivo fluorescent signal of different organs. **Figure S23.** Blood circulation half-life curve of PTZ-TQ-AIE dots in mice. The circulation half-life was determined to be 61 minutes by fitting the data (5 min, 10 min, 20 min, 0.5 h, 1 h, 3 h, 6 h, 9 h, 12 h, 24 h, 30 h) to a first-order exponential decay (n=3). **Figure S24.** Ex vivo NIR-II fluorescent images of main organs collected after 2 h post injection. **Figure S25.** The quantitative analysis of fluorescence intensity of orthotropic liver tumor at different time points after tail vein injection of PTZ-TQ-AIE dots (0.2 mL, 0.5 mg/mL). **Figure S26.** (a) Signal-to-noise ratio (SNR) and (b) tumor-normal liver ratio of Figure 4c (n=3). **Figure S27.** (a) Pictures of tumor resection process. (b) H&E staining of excised tumor and normal liver tissue (scale bar: 200 μm). **Figure S28.** (a) Bioluminescence imaging of orthotopic liver cancer mice treated with PBS, PBS+Laser, PTZ-TQ-AIE dots, PDT, Surgery, Surgery+PDT, PDT+Surgery (BT: Before PDT; AT: after PDT; BS: Before surgery; AS: after surgery). (b) body weight curves after different treatments (n=3). **Figure S29.** MR imaging of orthotopic liver cancer mice treated with PBS, PBS+Laser, PTZ-TQ-AIE dots, PDT, Surgery, Surgery+PDT, PDT+Surgery after therapy. **Figure S30.** H&E staining of heart, liver, spleen, lung, and kidney tissue slices for different groups after treatments: (a) PBS, (b) PBS+Laser, (c) PTZ-TQ-AIE dots, (d) PDT, (e) Surgery, (f) Surgery+PDT, (g) PDT+Surgery.

**Additional file 2.** Video of the NIR-II imaging guided surgery in orthotopic liver cancer model after tail vein injection of the PTZ-TQ-AIE dots 2 h and 48 h.

## Data Availability

All data analyzed during this study are included in this published article and its additional file.
