# Supplementary material for: NIR-II emissive AIEgen photosensitizers enable ultrasensitive imaging-guided surgery and phototherapy to fully inhibit orthotopic hepatic tumors
Source: J Nanobiotechnology. 2021 Dec 13;19:419. doi: 10.1186/s12951-021-01168-w (PMC8670198; doi:10.1186/s12951-021-01168-w)
Supplement: Supplementary file 1 — Additional file 1: Figure S1. Synthetic route of the PTZ-TQ molecule. Figure S2. 1H NMR spectrum of 3 in CDCl3. Figure S3. 13C NMR spectrum of 3 in CDCl3. Figure S4. 1H NMR spectrum of 4 in DMSO-d6. Figure S5. 13C NMR spectrum of 4 in DMSO-d6. Figure S6. 1H NMR spectrum of 5 in DMSO-d6. Figure S7. 13C NMR spectrum of 5 in DMSO-d6. Figure S8. 1H NMR spectrum of 6 in DMSO-d6. Figure S9. 13C NMR spectrum of 6 in DMSO-d6. Figure S10. 1H NMR spectrum of PTZ-TQ in pyridine-d5. Figure S11. 13C NMR spectrum of PTZ-TQ in pyridine-d5. Figure S12. The mass spectrum of PTZ-TQ. m/z: calcd. 958.39, found: 959.39 for [M]+. Figure S13. The zeta potentials of PTZ-TQ and PTZ-TQ-AIE dots. Figure S14. The stability evaluation of PTZ-TQ-AIE dots in PBS based on hydrodynamic size. PTZ-TQ-AIE dots were incubated in PBS at different time points. The hydrodynamic size at 0 h, 2 h, 4 h, 8 h, 12 h, 24 h, 48 h and 72 h, had no obvious changes compared with 0 h. Figure S15. The stability evaluation of PTZ-TQ-AIE dots in DMEM based on hydrodynamic size (a-d). PTZ-TQ-AIE dots were incubated in DMEM at different time points. The hydrodynamic size at 0 h (a), 12 h (b), 24 h (c), 48 h (d) had no obvious changes compared with 0 h. Figure S16. The stability evaluation of PTZ-TQ-AIE dots in 5% FBS based on hydrodynamic size (a-d). PTZ-TQ-AIE dots were incubated in 5% FBS at different time points. The hydrodynamic size at 0 h (a), 12 h (b), 24 h (c), 48 h (d) had no obvious changes compared with 0 h. Figure S17. Fluorescence quantum yield measurements of PTZ-TQ-AIE dots in water. Absorbance and fluorescence spectra of IR26 in DEM (a-c), and PTZ-TQ-AIE dots in water (d-f). The integrated fluorescence was plotted against absorbance for both IR26 and fluorophores and fitted into a linear function, linear fit of IR26 (c) and PTZ-TQ-AIE dots (f). Figure S18. (a) ROS generation of PTZ-TQ-AIE dots with different concentrations. (b) ROS generation of PTZ-TQ-AIE dots with different times. The light source: 808 n [file 12951_2021_1168_MOESM1_ESM.docx]

**NIR-II emissive AIEgen photosensitizers enable ultrasensitive imaging-guided surgery and phototherapy to fully inhibit orthotopic hepatic tumors**

Ruizhen Jia, Han Xu, Chenlu Wang, Lichao Su, Jinpeng Jing, Shuyu Xu, Yu Zhou, Wenjing Sun, Jibin Song, Xiaoyuan Chen, Hongmin Chen*

R. Jia, H. Xu, J. Jing, S. Xu, Prof. H. Chen

State Key Laboratory of Molecular Vaccinology and Molecular Diagnostics & Center for Molecular Imaging and Translational Medicine, School of Public Health, Xiamen University, Xiamen 361102, China

E-mail: hchen@xmu.edu.cn

C. Wang, L. Su, Prof. J. Song

MOE Key Laboratory for Analytical Science of Food Safety and Biology, College of Chemistry, Fuzhou University, Fuzhou 350108, China

Y. Zhou

State Key Laboratory of Physical Chemistry of Solid Surfaces, College of Chemistry and Chemical Engineering, Xiamen University, Xiamen361005, China

Prof. X. Chen

Departments of Diagnostic Radiology and Surgery, Clinical Imaging Research Centre, Centre for Translational Medicine, Nanomedicine Translational Research Program, NUS Center for Nanomedicine, Yong Loo Lin School of Medicine, Departments of Chemical and Biomolecular Engineering, and Biomedical Engineering, Faculty of Engineering, National University of Singapore, Singapore

R. Jia, H. Xu, C. Wang contributed equally to this work.

**Experimental section**

**Synthesis and characterization of PTZ-TQ.** 1-Bromooctane, 10H-phenothiazine, sodium hydrid, NH_4_Cl，anhydrous Na_2_SO_4_, N-bromosuccinimide and 2-isopropoxy- 4,4,5,5-tetramethyl-1,3,2-dioxaborolane were obtained from Meryer (Shanghai) Chemical Technology Co., Ltd. dry dichloromethane. trifluoromethanesulfonic acid, 4,7-dibromobenzo[c][1,2,5] thiadiazole, iron powder, acetic acid and benzil were obtained from Zhengzhou aikemu Chemical Co., Ltd., mPEG-DSPE-NH_2_ (3400) were obtained from Shanghai Yare Biotech, Co., Ltd. PBS were obtained from HyClone Co., Ltd. dry N,N-dimethylformamide(DMF) and dry tetrahydrofuran(THF) All reagents were obtained from Energy Chemical Co., Ltd and used without further purification, except if stated otherwise. 1H spectra were measured on a 400 MHz ZhongKe-NiujinAS400 spectrometer The UV-vis-NIR spectrum were obtained on a spectrophotometer (Lambda950, America), and fluorescence spectrum were obtained with fluorometer FLS980 (Edinburgh Instruments, England). The MS spectra of the compound was measured by LC-MS (Thermo Q-Exactive,). The size of PTZ-TQ-AIE dots was measured by transmission electron microscope: (Tecnai Spirit T12) and Zetasizer Nano ZS90: Malvernv Instruments Co., Ltd. The MTT assay of the PTZ-TQ-AIE dots was measured by Enzyme-labelled meter: BioTek CO., Ltd (USA). The fluorescence images of cells were taken on a laser scanning confocal microscopy (Olympus FV1200, Japan). Ltd.

**Synthesis of compound 1**

7 mL of fuming nitric acid was added dropwise into 100 g of trifluoromethanesulfonic acid in ice water bath and the resulting mixture was further stirred for 1 h. Then 4,7-dibromobenzo[c][1,2,5] thiadiazole (12 g, 40.8 mmol) was added into the mixture portion-wise over 1 h. Thereafter, the resulting mixture was stirred at 55 °C for another 48 h and poured into 500 mL ice-water mixture after cooled to room temperature. The produced precipitate was filtered and washed with water, then purified by column chromatography on silica gel (hexane / DCM (V / V) = 1/ 2) to obtain compound **1** as a yellow solid (2.2 g, 14 %)

**Synthesis of compound 2**

Compound **1** (2.01 g, 5.22 mmol) and iron powder (2.81 g, 50.2 mmol) were dispersed in 70 mL of acetic acid. The mixture was stirred at 80 °C for 6 h and poured into 200 mL ice-water mixture after cooled to room temperature. The produced precipitate was filtered and washed with water for three times, then dried to obtain compound **2** as a yellow powder (1.56 g, 82 %).

**Synthesis of compound 3**[**^1^**](#_ENREF_1)

Compound **2** (2 g, 6.2 mmol) and benzil (1.32 g, 6.31 mmol) were dispersed in 90 mL of acetic acid and stirred at 100 °C for 72 h. After cooling down, the reaction mixture was diluted with water and extracted by chloroform for three times. The combined organic phase was washed with saturated aqueous NaHCO_3_ and dried with anhydrous MgSO_4_. After the removal of the solvent, the residue was purified by column chromatography on silica gel to obtain compound **3** as an orange-red solid (1.2 g, 36.6 %)

^1^H NMR (400 MHz, *CDCl_3_*) δ: 7.81 (d, J = 7.5 Hz, 4H), 7.55-7.33 (m, 6H)

^13^C NMR (100 MHz, *CDCl_3_*) δ: 155.99, 152.38, 138.08, 137.60, 130.37, 13025, 128.40, 114.20.

**Synthesis of compound 4**

A solution of 10H-phenothiazine (20 g, 100 mmol) in 50 mL DMF was added dropwise into sodium hydride (6 g, 150 mmol, 60 % in mineral oil) in an ice-water bath over 1 h. Thereafter, a solution of 1-bromohexane (25.70 g, 133.3 mmol) in 10 mL DMF was added dropwise into the mixture over 30 minutes. The resulting mixture was stirred at room temperature for 36 h. After completion of the reaction, the mixture was added saturated aqueous NH_4_Cl and extracted with dichloromethane for three times. The combined organic phase was dried with anhydrous Na_2_SO_4_ and removed the solvent, the residue was purified by column chromatography on silica gel (hexane) to obtain compound 4 as a light-yellow oil. (21.3 g, 71.92 %).

^1^H NMR (400 MHz, *DMSO-d_6_*) δ: 7.23-7.17 (m, 2H), 7.15 (dd, J = 7.7, 1.5 Hz, 2H), 6.99 (dd, J = 8.3, 1.2 Hz, 2H), 6.96-6.91(m, 2H), 3.86 (t, J = 7.0 Hz, 2H), 1.72-1.66 (m, 2H), 1.41-1.31 (m, 2H), 1.29-1.12 (m, 8H), 0.83-0.80 (m, 3H).

^13^C NMR (400 MHz, *DMSO-d_6_*) δ: 145.28, 127.93, 127.60, 124.22, 122.78, 116.21, 46.91, 31.66, 29.05, 28.98, 26.70, 26.68, 22.48, 14.33.

**Synthesis of compound 5**

A solution of the compound **4** (10.0 g, 33.57 mmol) in 120 mL of dry dichloromethane was stirred in an ice-water bath for 15 min and N-bromosuccinimide (3.0 g, 16.85 mmol) was added to the solution portion-wise over 1.5 h under dark condition. The resulting mixture was stirred at room temperature overnight. After completion of the reaction, the mixture was diluted with 100 mL water and extracted with dichloromethane for three times. The combined organic phase was dried with anhydrous Na_2_SO_4_ and removed the solvent, the residue was purified by column chromatography on silica gel (hexane) to obtain compound **5** as a light-yellow oil (3.1 g, 23.61%).

^1^H NMR (400 MHz, *DMSO-d_6_*) δ: 7.35 -7.29 (m, 2H), 7.23-7.17 (m, 1H), 7.13 (dd, J = 7.6, 1.6 Hz, 1H), 7.01-6.89 (m, 3H), 3.83 (t, J = 6.9 Hz, 2H), 1.69-1.57 (m, 2H), 1.39-1.28 (m, 2H), 1.25-1.17 (m, 8H), 0.83-0.76 (m, 3H).

^13^C NMR (400 MHz, *DMSO-d_6_*) δ: 144.86, 144.65, 130.42, 129.30, 128.18, 127.69, 126.81, 123.43, 122.11, 117.78, 116.40, 114.06, 47.02, 31.06, 29.03, 28.96, 26.66, 26.49, 22.47, 14.31.

**Synthesis of compound 6**

Slowly dropped n-butyllithium (1.6 M in hexane, 5.0 mL, 8.12 mmol) to a solution of compound **5** (2.54 g, 6.49 mmol) in 20 mL of dry THF at -78 ^o^C with argon shield, the resulting mixture was stirred for 1 h. Thereafter, 2-isopropoxy- 4,4,5,5-tetramethyl-1,3,2-dioxaborolane (1.6 g, 8.69 mmol) was added at -78 ^o^C and the mixture was stirred for another 1 h, then warmed to room temperature and stirred overnight. After completion of the reaction, the mixture was added 50 mL of saturated aqueous NH_4_Cl and extracted by dichloromethane for three times. The combined organic phase was dried with anhydrous Na_2_SO_4_ and removed the solvent, the residue was purified by column chromatography on silica gel (hexane / ethyl acetate (V / V) = 50 / 1) to obtain compound **6** as a viscous yellow oil (446 mg, 16.2%).

^1^H NMR (400 MHz, *DMSO-d_6_*) δ: 7.62-7.49 (dd, J = 8.1, 1.5 Hz, 1H), 7.33 (d, J = 1.4 Hz, 1H), 7.26-7.18 (m, 2H), 7.02-6.97 (m, 2H), 6.96-6.91 (m, 3H), 3.85 (t, J = 6.9 Hz, 2H), 1.70-1.60 (m, 2H), 1.42-1.30 (m, 2H), 1.26 (s, 12H), 1.24-1.19 (m, 8H), 0.83-0.78 (m, 3H).

^13^C NMR (100 MHz, *DMSO-d_6_*) δ: 147.93, 144.59, 136.30，134.61, 133.33，128.03, 127.54, 123.38, 122.22, 120.19, 116.46, 115.74, 83.97, 83.74，46.92, 31.51, 28.88, 26.55, 25.07, 22.44, 14.34.

**Synthesis of compound PTZ-TQ**

A 50 mL Schlenk flask was charged with compound **6** (160 mg, 0.377 mmol), compound **3** (62 mg, 0.125 mmol), SPhos (2-dicyclohexylphosphino-2',6'-dimethoxybiphenyl) (21 mg, 0.051 mmol), anhydrous K_3_PO_4_ (105 mg, 0.495 mmol), 2 mL of dry THF, 0.2 mL of deionized water and palladium (II) acetate (5 mg, 0.022 mmol). The resulting mixture was stirred at 85 °C with argon shield for 30 h. After completion of the reaction, the mixture was diluted with 10 mL of water and extracted with dichloromethane for three times. The combined organic phase was dried with anhydrous MgSO_4_ and removed the solvent, the residue was purified by column chromatography on silica gel (DCM / PE (V/V) = 1 / 1) to obtain compound PTZ-TQ as a blue solid (37mg, 30.83%).

^1^H NMR (400 MHz, *pyridine-d_5_*) δ: 8.45-8.36 (m, 4H), 8.05-7.97 (m, 4H), 7.46-7.41 (m, 10H), 7.39-7.30 (m, 2H), 7.17-7.09 (m, 5H), 4.07-3.96 (m, 4H), 1.96-1.83 (m, 4H), 1.54-1.47 (m, 4H), 1.37-1.19 (m, 16H),0.95-0.87 (m,6H).

^13^C NMR (100 MHz, *pyridine-d_5_*) δ: 153.86,146.27, 145.82, 139.76, 136.83,135.66, 133.83, 133.20, 131.03, 130.25, 130.20, 129.02, 128.64, 128.34, 125.26, 124.04, 123.66, 116.79, 115.64, 48.11, 32.38, 29.95, 29.89, 27.70,27.60,23.28,14.68.

**Preparation of PTZ-TQ-AIE dots**[**^2^**](#_ENREF_2)

A THF solution (1 mg/mL, 1 mL) of PTZ-TQ and ultrapure water (1 mg/mL, 6 mL) of DSPE-PEG-NH_2_ 3400) were added into a 25 mL-round-bottom flask, The mixture was stirred for 24 h, and centrifuged to remove the solvents with an ultrafiltration tube. The obtained PTZ-TQ-AIE dots were then diluted with a certain amount of PBS buffer (pH = 7.4) to get the desired concentration for further studies.

**Determination of QYs**[**^3-4^**](#_ENREF_3)

A series of the solutions of the **PTZ-TQ-AIE dots** and the reference dye were prepared at different concentrations, respectively. Their absorption and emission spectra were measured and their QY were calculated by the following formula:

𝑄𝑌_𝑠𝑎𝑚𝑝𝑙𝑒_ = 𝑄𝑌_𝐼𝑅26_ ×Slope_sample_/ Slope_IR26_×n_sample_^2^/ n_IR26_^2^

Where QY_sample_ is the QY of fluorophore **PTZ-TQ-AIE dots** in water, QY_IR26_ is the QY of IR-26 in DCE, n_sample_ and n_IR26_ are the refractive indices of corresponding solvents and DCE.

**Cytotoxicity**

HepG2 cells and LO2 cells were seeded in a 96-well plate at a density of 1×10^4^ cells/100 μL and incubated for 24 h in DMEM medium supplemented with 10 % of fetal bovine serum (FBS), 100 units/mL of penicillin and 100 mg/mL of streptomycin at 37 °C in a humidified atmosphere of 5 % CO_2_. Then the cells were treated with PTZ-TQ-AIE dots at different concentrations and co-incubated for another 24 h. 5.0 mg/mL stock solution of MTT was prepared in PBS and this stock solution (10 μL) was added to each well. After additional 4.0 h incubation, the medium and MTT were removed, and the MTT-formazan crystals in each well were dissolved in 150 μL of DMSO. The absorbance of the suspension was recorded by a microplate reader at wavelength of 490 and 570 nm.

The phototoxicity of PTZ-TQ-AIE dots was also evaluated. The HepG2 cells cultured in 96-well plates as described above were incubated with PTZ-TQ-AIE dots at different concentrations (5, 10, 25, 50 and 100 µg/mL) for 6 h. Then the cells were irradiated by an 808 nm NIR laser at a power density of 0.25 W/cm^2^ for 10 min. The area of each well was fully covered by the laser spot. After being illuminated, the cells were incubated for another 24 h before the MTT assay. The cell viability was normalized to the control group without any treatment.

**ROS generation study**

The fluorescent probe DCFH-DA was applied as indicator to detect the ROS generation efficiency. The fluorescence intensity of DCFH-DA enhanced after reaction with ROS. In brief, the activated DCFH-DA (5 × 10^-6^ M) solution was mixed separately with different samples, Then the mixture was irradiated with 808 nm NIR laser irradiation (NIR, 0.25 W/cm^2^) for 10 min, and determined via fluorescent spectrophotometer (excitation wavelength: 488 nm and emission wavelength: 525 nm).

**Generation of intracellular ROS**

The generation of intracellular ROS was evaluated in living HepG2 cells using DCFH-DA as indicator. HepG2 cells were seeded into confocal plates and incubated for 24 h. The culture mediums were firstly replaced with 1 mL fresh medium containing PTZ-TQ-AIE dots (50 μg/mL), the cells were incubated for another 4 h at 37 ^o^C. Then the cells were washed with PBS and incubated with 0.5 mL fresh medium containing 5 × 10^-6^ M DCFH-DA for additional 20 min at 37 ^o^C. The cells were washed with PBS and irradiated by 808 nm NIR laser (0.25 W/cm^2^) for 10 min. After that, the cells were washed with PBS and stained with Hoechst 33342 for 15 min at 37 ^o^C. Finally, the fluorescence images of cells were captured by CLSM. The exciting wavelength and emission filter of DCFH-DA were 488 and 500-550 nm, respectively. Scale bar: 20 μm.

**Animals**

All the animal experimental procedures were approved by the Institutional Animal Care and Use Committee of Xiamen University. Mice, weighing approximately 20 g, were used for assessing all treatment. Briefly, mice were anesthetized, and then 25 µL HepG2 cells per mouse) were injected into the right liver lobe by a laparotomy. On the two weeks after the operation, bioluminescence imaging performed with IVIS Lumina II after intraperitoneal injection of fluorescein substrate to screen out tumor-bearing mice for NIR-II imaging and treatment.[^5-6^](#_ENREF_5)

**In vivo pharmacokinetics and biodistribution evaluation.**

To investigate the pharmacokinetics of PTZ-TQ-AIE dots, the dots was intravenously injected into Babl/c male mice (n = 3). The blood samples were collected at different time points from 1 min to 48 h. The time-dependent relative fluorescent intensity in blood were fitted with a first-order exponential decay to evaluate the blood half-life. The biodistributions of the PTZ-TQ-AIE dots in mice were also investigated by sacrificing the mice after 168 h injection. The main organs (including the heart, liver, spleen, lung, kidney) were collected for *ex vivo* NIR-II bioimaging by using the same imaging system.

**In Vivo NIR-II Fluorescence Imaging**

All NIR-II fluorescent images were collected using a NIR-II imaging system with the indium-gallium-arsenide (InGaAs) camera (Princeton Instruments). The excitation light source was an 808 nm diode laser. The mice were anesthetized by intraperitoneal injection of pentobarbital sodium solution (0.2 mL, 500 μg/mL) during Surgery and NIR-II imaging. For tumor and tumor-feeding blood vessels imaging, the BALB/c mice bearing subcutaneous an orthotropic liver tumor model was given PTZ-TQ-AIE dots (0.2 mL, 500 μg/mL) via tail vein injection. After injection, the mice were mounted in the prone position beneath the laser for imaging at various time points. For hind limb vasculature imaging, PTZ-TQ-AIE dots (0.2 mL, 500 μg/mL) were given to normal mice via the tail vein injection.

**NIR-II optical-guided tumor resection**

The colorectal tumor-bearing nude mice were intravenously injected with PTZ-TQ-AIE dots (0.2 mL, 500 μg/mL). The tumors can be clearly distinguished from the normal tissues after 2 h and 48 h post-injection. Then the tumors were resected from the nude mice under real-time NIR-II optical bioimaging guidance and analyzed by (hematoxylin and eosin) H&E staining.

**Statistical analysis**

Statistical analyses were carried out using independent t-tests. All data are presented as mean ± standard error. Statistical significance is defined as *: P < 0.05, **: P < 0.01, and ***: P < 0.001.

**References**

1. Li, S.; Yin, C.; Wang, R.; Fan, Q.; Wu, W.; Jiang, X., Second Near-Infrared Aggregation-Induced Emission Fluorophores with Phenothiazine Derivatives as the Donor and 6, 7-Diphenyl-[1, 2, 5] Thiadiazolo [3, 4-g] Quinoxaline as the Acceptor for In Vivo Imaging. *ACS Appl. Mater. Interfaces.* **2020**, 12 (18), 20281-20286.

2. Lin, J.; Zeng, X.; Xiao, Y.; Tang, L.; Nong, J.; Liu, Y.; Zhou, H.; Ding, B.; Xu, F.; Tong, H., Novel near-infrared II aggregation-induced emission dots for in vivo bioimaging. *Chem. Sci.* **2019**, 10 (4), 1219-1226.

3. Semonin, O. E.; Johnson, J. C.; Luther, J. M.; Midgett, A. G.; Nozik, A. J.; Beard, M. C., Absolute photoluminescence quantum yields of IR-26 dye, PbS, and PbSe quantum dots. *J. Phys. Chem. Lett.* **2010**, 1 (16), 2445-2450.

4. Xu, Y.; Zhang, Y.; Li, J.; An, J.; Li, C.; Bai, S.; Sharma, A.; Deng, G.; Kim, J. S.; Sun, Y., NIR-II emissive multifunctional AIEgen with single laser-activated synergistic photodynamic/photothermal therapy of cancers and pathogens. *Biomaterials*. **2020**, 259, 120315.

5. Shi, X.; Ma, X.; Ren, E.; Zhang, Y.; Jia, D.; Gao, Y.; Xue, P.; Kang, Y.; Liu, G.; Xu, Z., Tumor-microenvironment-activatable nanoreactor based on a polyprodrug for multimodal-imaging-medicated enhanced cancer chemo/phototherapy. *ACS Appl. Mater. Interfaces.* **2019**, 11 (43), 40704-40715.

6. Shi, T.; Sun, W.; Qin, R.; Li, D.; Feng, Y.; Chen, L.; Liu, G.; Chen, X.; Chen, H., X‐Ray‐Induced Persistent Luminescence Promotes Ultrasensitive Imaging and Effective Inhibition of Orthotopic Hepatic Tumors. *Adv. Funct. Mater*. **2020**, 30 (24), 2001166.


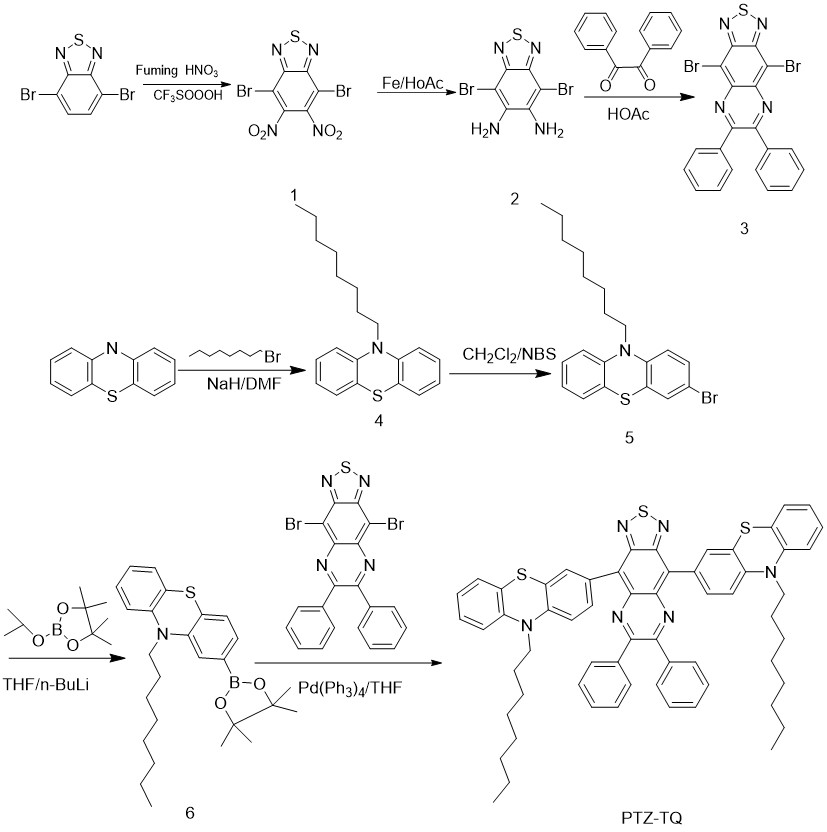


**Figure S1**. Synthetic route of the PTZ-TQ molecule.

**
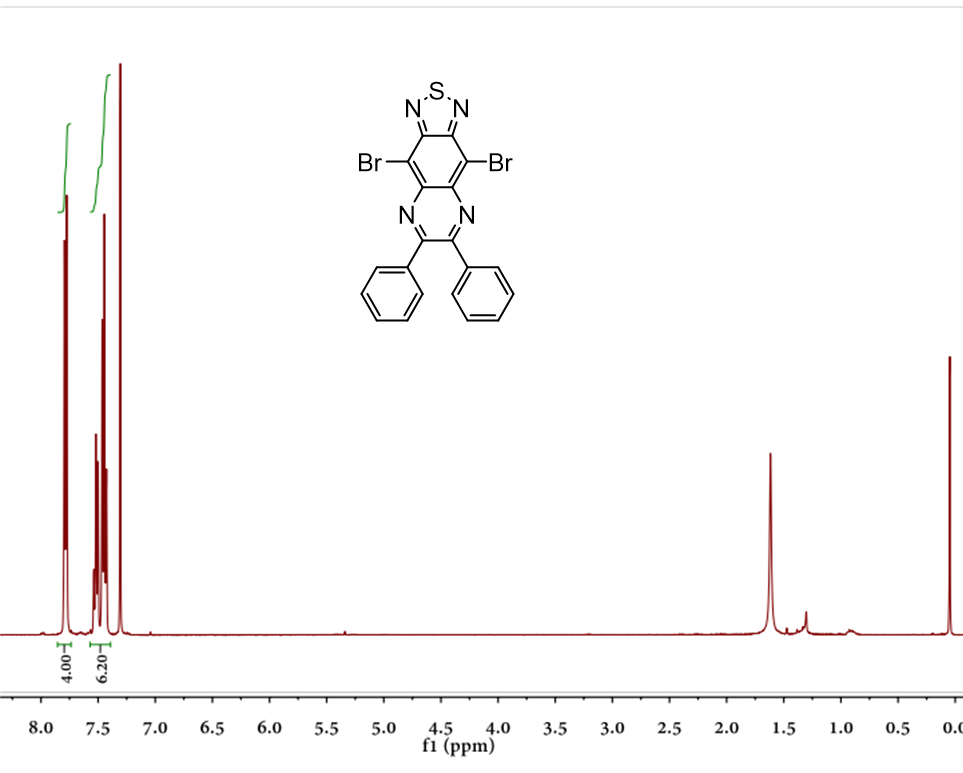
**

**Figure S2**. ^1^H NMR spectrum of **3** in CDCl_3_.


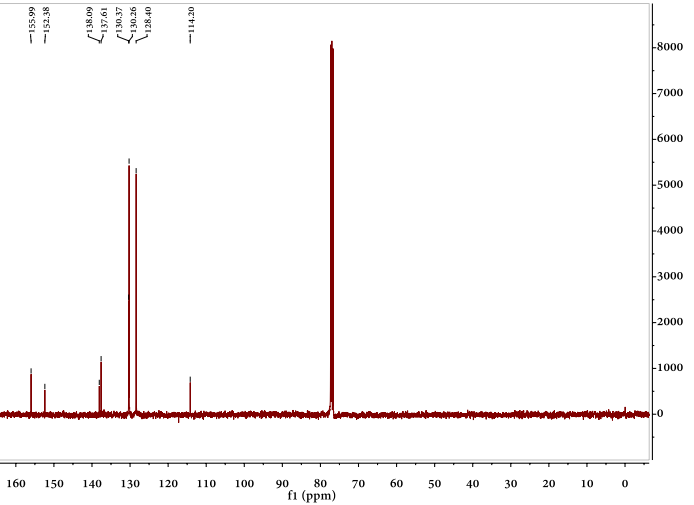


**Figure S3**. ^13^C NMR spectrum of **3** in CDCl_3_.


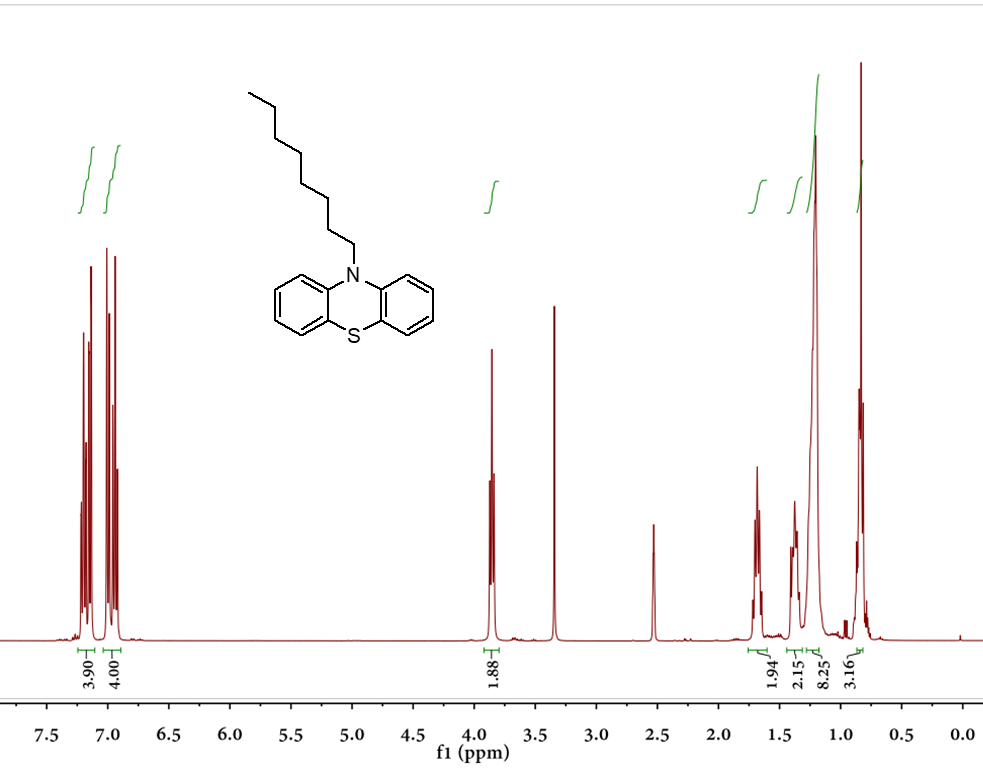


**Figure S4**. ^1^H NMR spectrum of **4** in DMSO-*d_6_*.


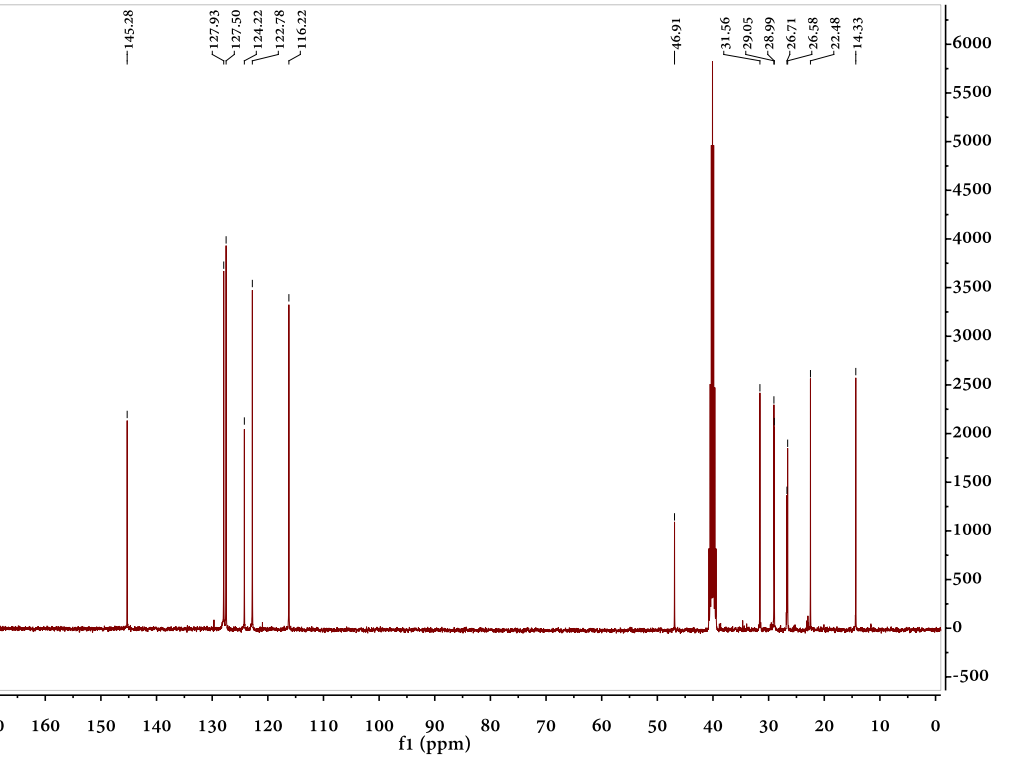


**Figure S5**. ^13^C NMR spectrum of **4** in DMSO-*d_6_*.

**
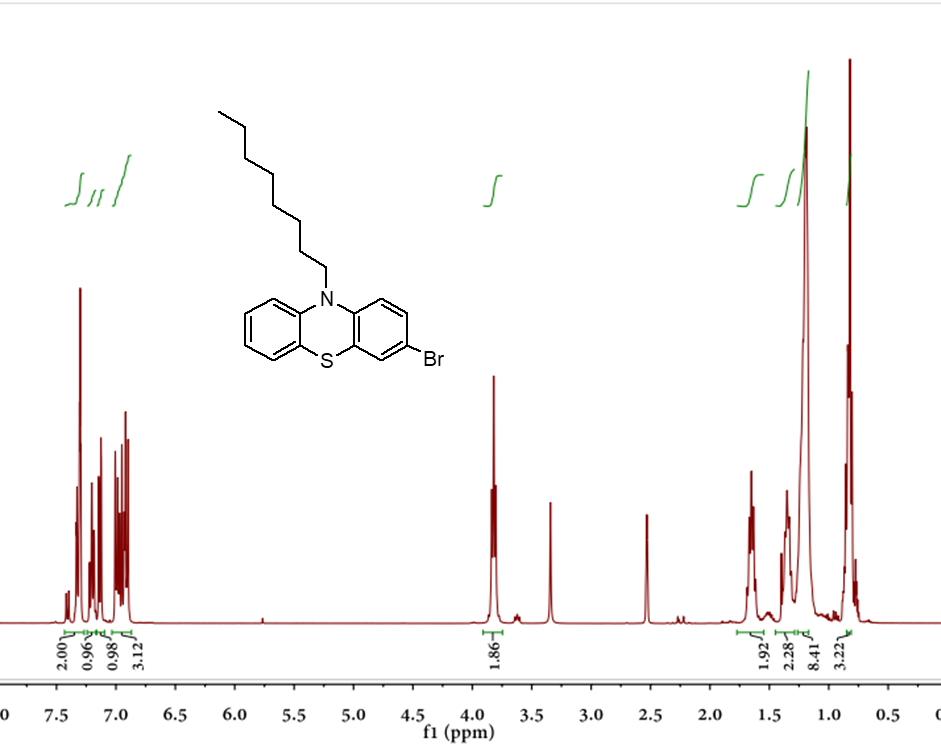
**

**Figure S6**. ^1^H NMR spectrum of **5** in DMSO-d_6_.


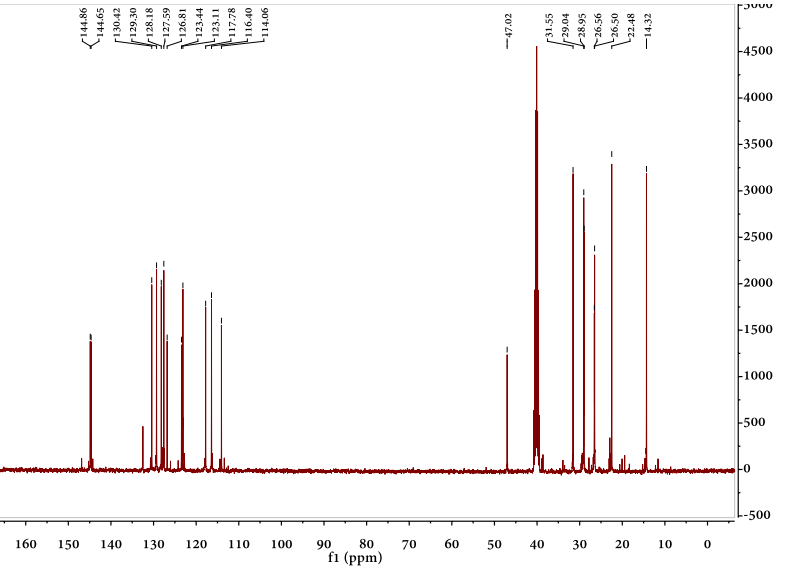


**Figure S7.** ^13^C NMR spectrum of **5** in DMSO-*d_6_*.

**
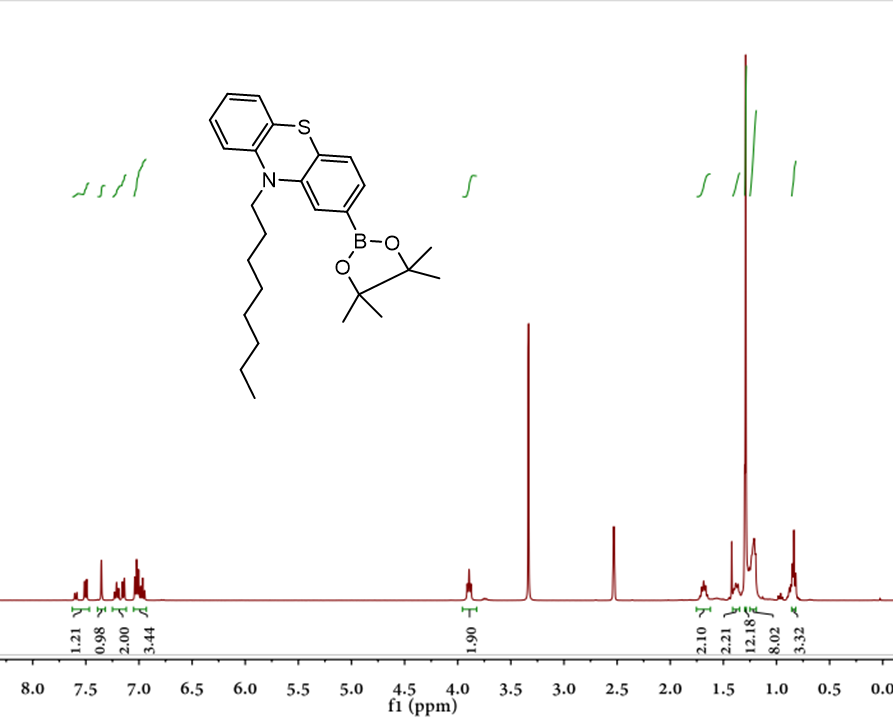
**

**Figure S8**. ^1^H NMR spectrum of **6** in DMSO-*d_6_.*


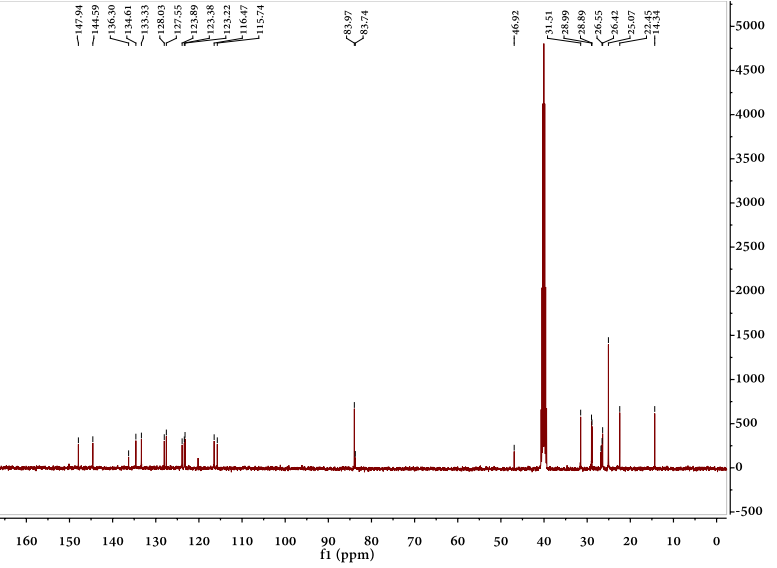


**Figure S9**. ^13^C NMR spectrum of **6** in DMSO-*d_6_*.


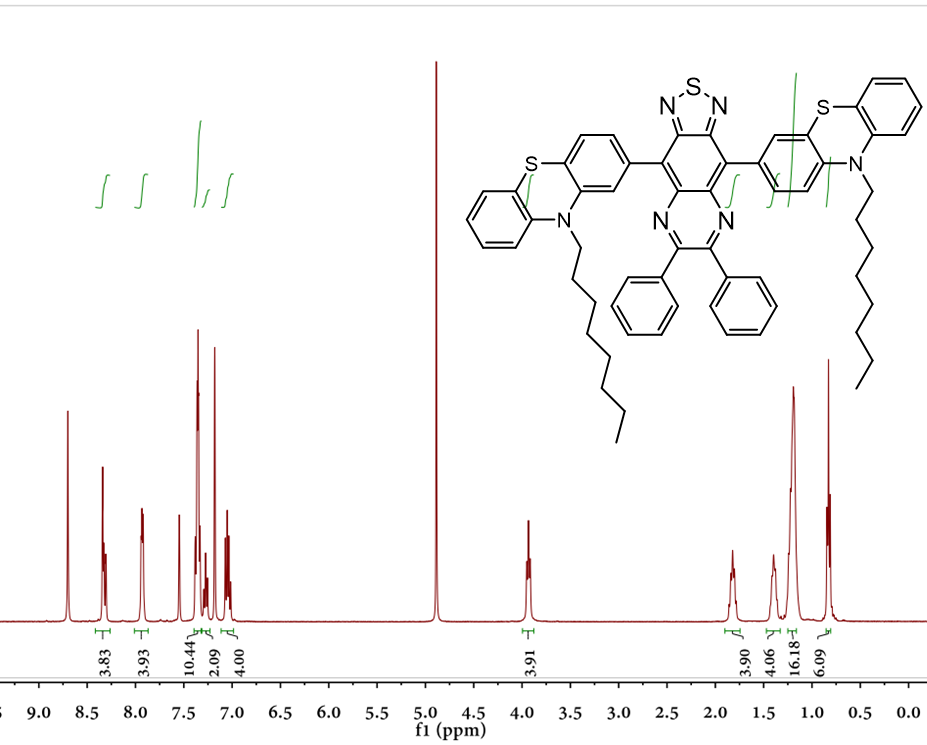


**Figure S10**. ^1^H NMR spectrum of **PTZ-TQ** in pyridine*-d_5_*.


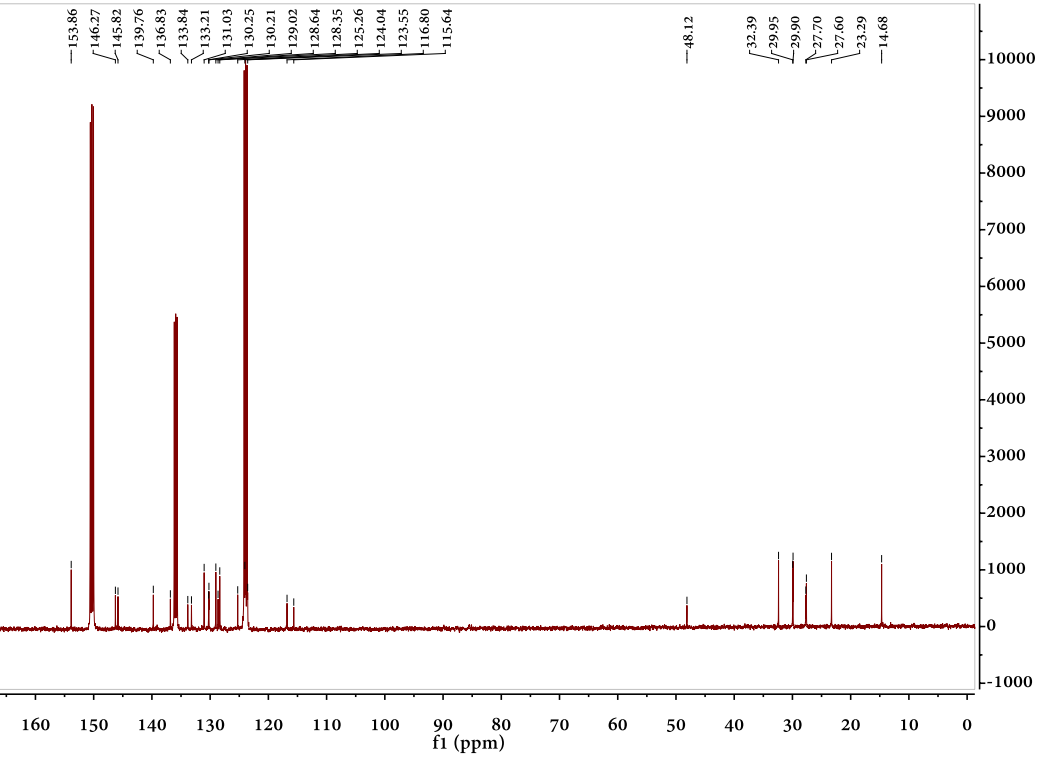


**Figure S11**. ^13^C NMR spectrum of **PTZ-TQ** in pyridine-*d_5_*_._


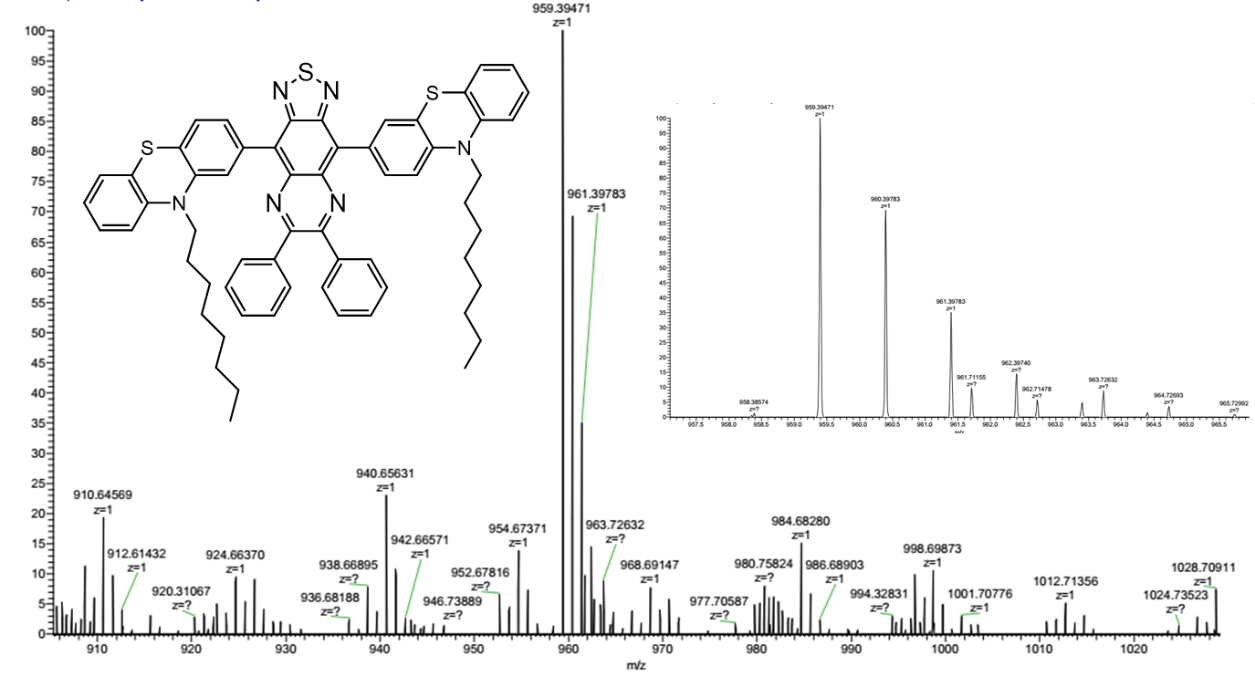


**Figure S12.** The mass spectrum of **PTZ-TQ**. *m/z*: calcd. 958.39, found: 959.39 for [M]^+^.

**Figure S13.** The zeta potentials of PTZ-TQ and PTZ-TQ-AIE dots.


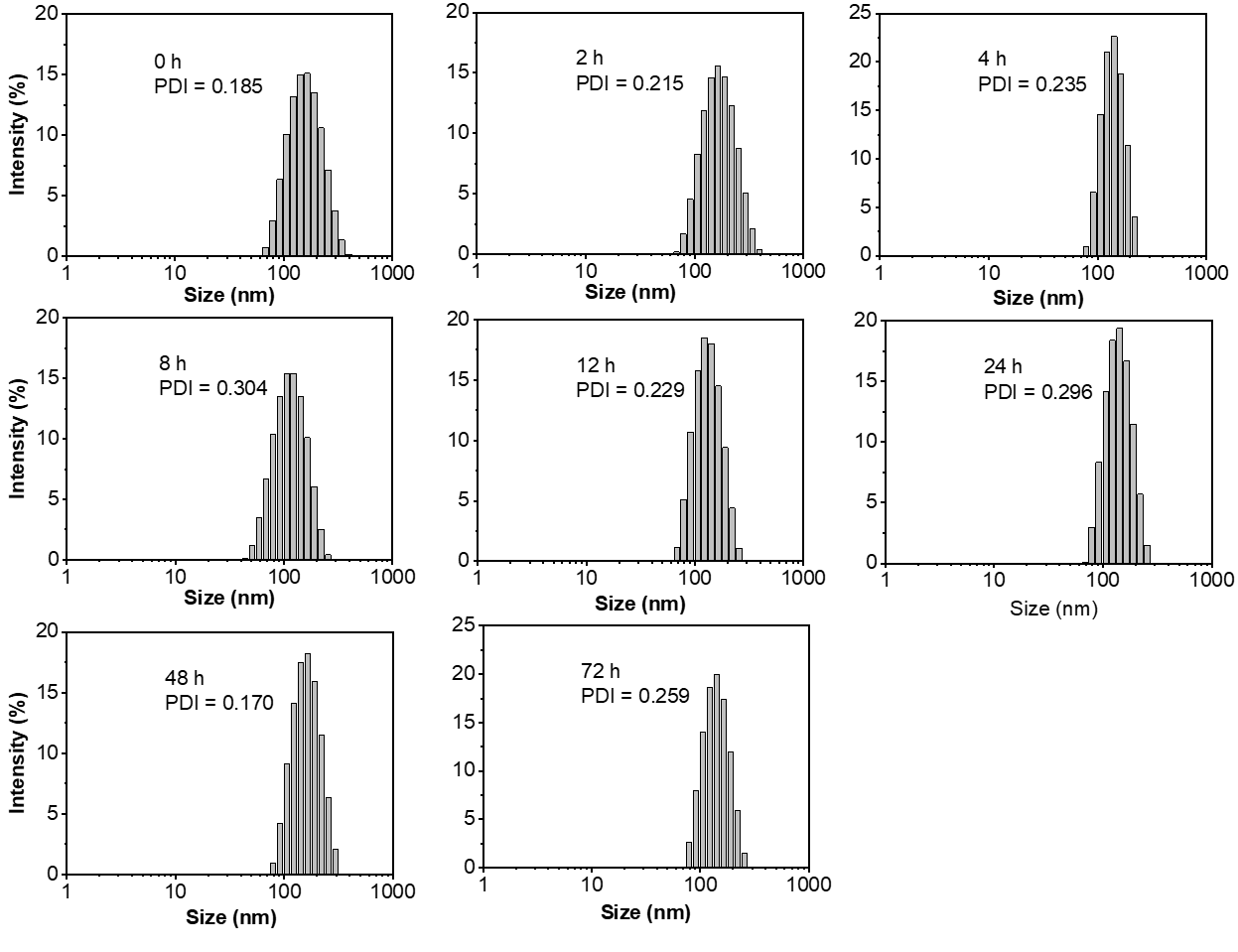


**Figure S14.** The stability evaluation of PTZ-TQ-AIE dots in PBS based on hydrodynamic size. PTZ-TQ-AIE dots were incubated in PBS at different time points. The hydrodynamic size at 0 h, 2 h, 4 h, 8 h, 12 h, 24 h, 48 h and 72 h, had no obvious changes compared with 0 h.


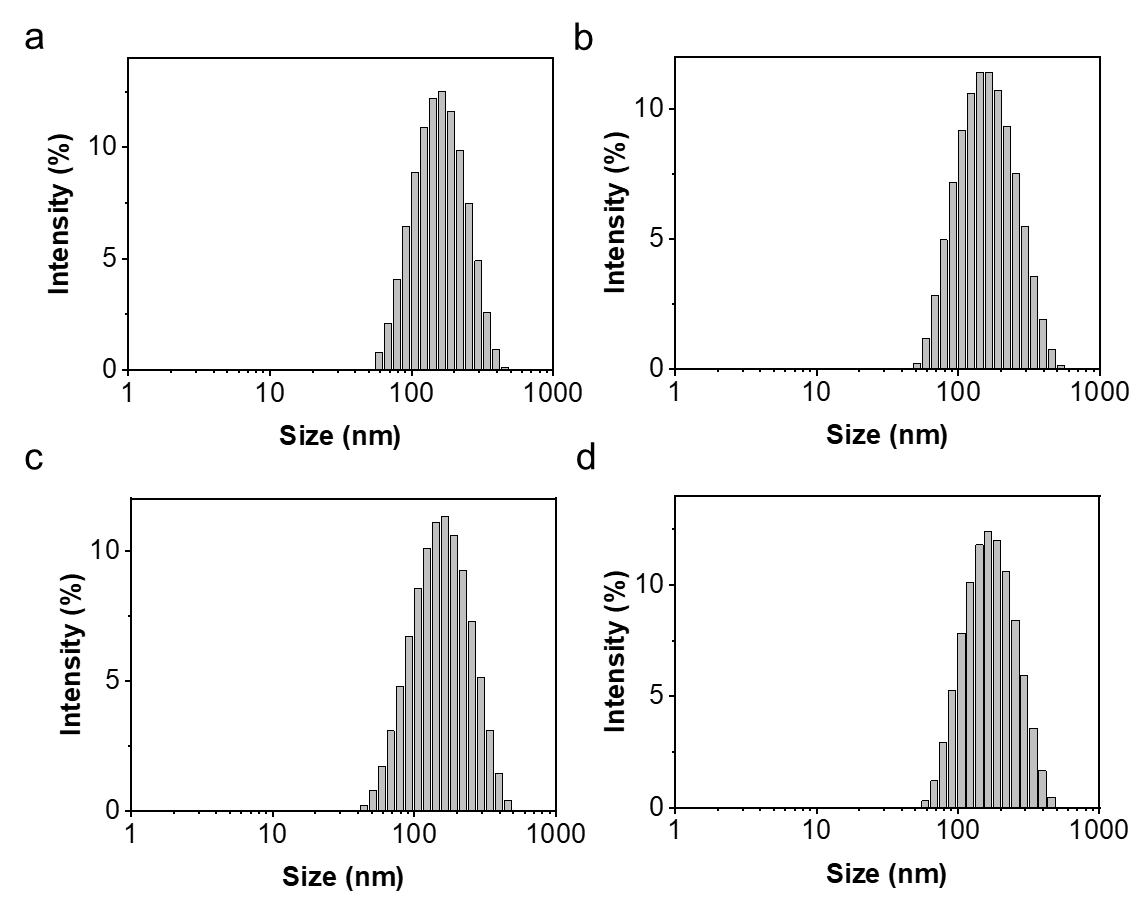


**Figure S15.** The stability evaluation of PTZ-TQ-AIE dots in DMEM based on hydrodynamic size (a-d). PTZ-TQ-AIE dots were incubated in DMEM at different time points. The hydrodynamic size at 0 h (a), 12 h (b), 24 h (c), and 48 h (d) had no obvious changes compared with 0 h.


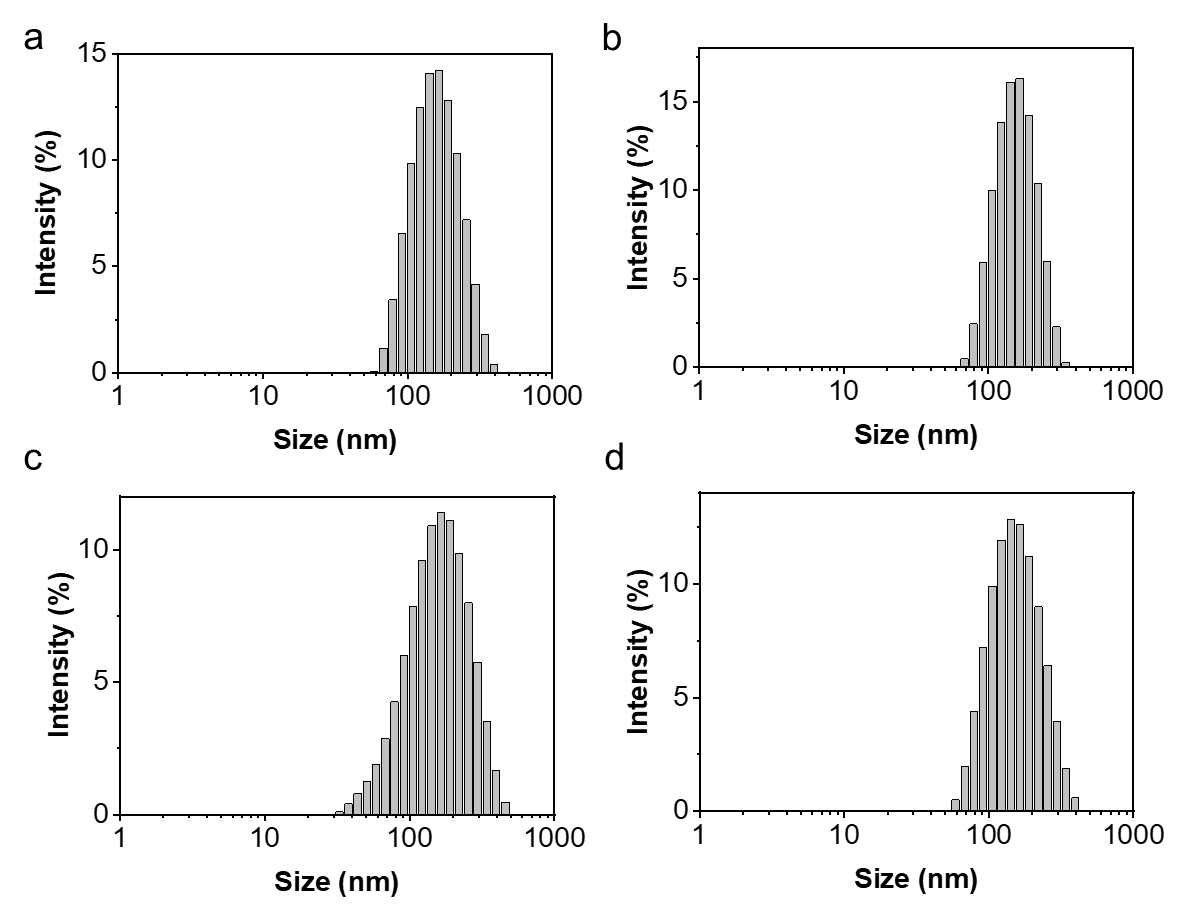


**Figure S16.** The stability evaluation of PTZ-TQ-AIE dots in 5% FBS based on hydrodynamic size (a-d). PTZ-TQ-AIE dots were incubated in 5% FBS at different time points. The hydrodynamic size at 0 h (a), 12 h (b), 24 h (c), and 48 h (d) had no obvious changes compared with 0 h.


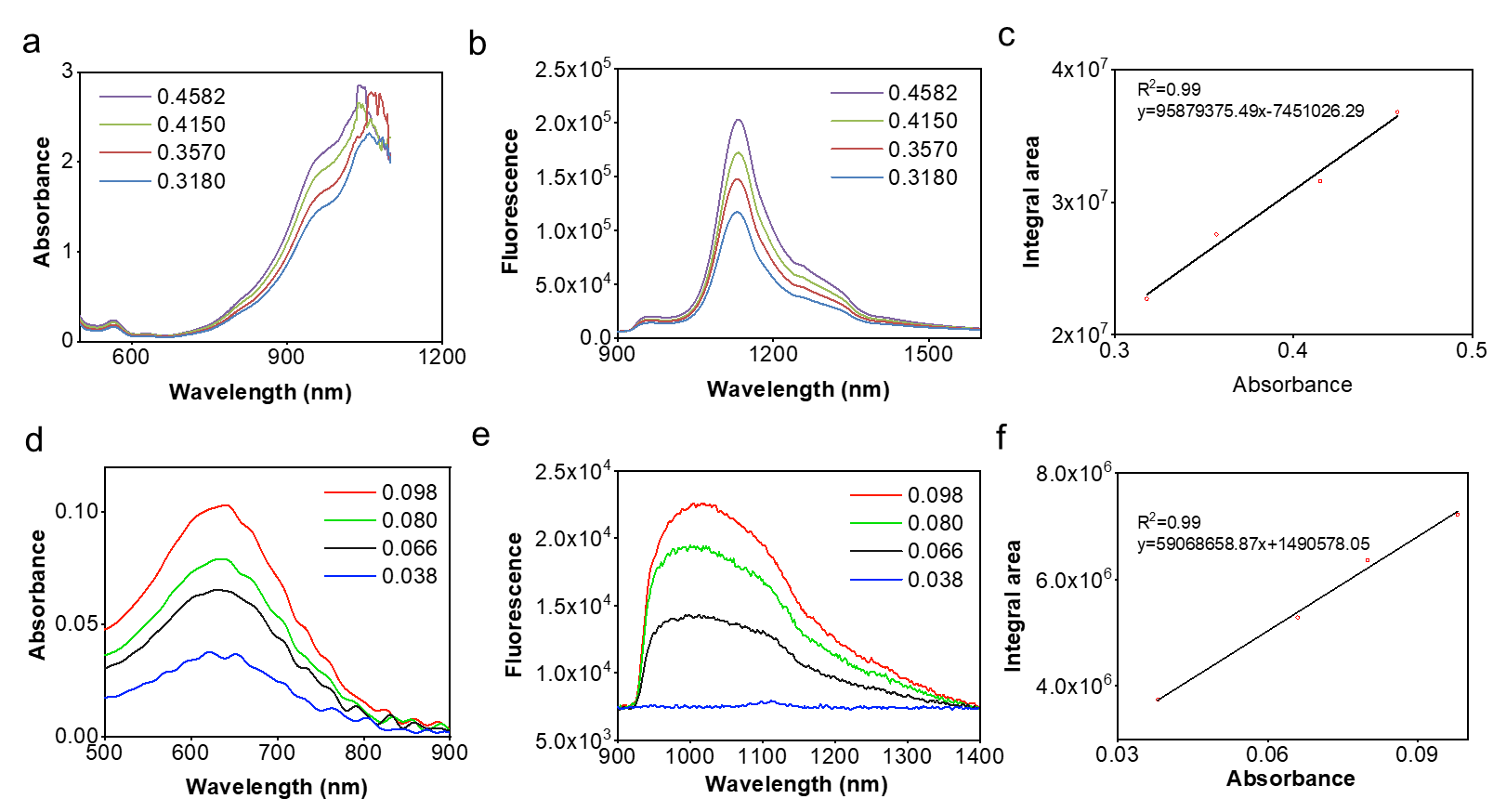


**Figure S17.** Fluorescence quantum yield measurements of PTZ-TQ-AIE dots in water. Absorbance and fluorescence spectra of IR26 in DEM (a-c), and PTZ-TQ-AIE dots in water (d-f). The integrated fluorescence was plotted against absorbance for both IR26 and fluorophores and fitted into a linear function, linear fit of IR26 (c) and PTZ-TQ-AIE dots (f).


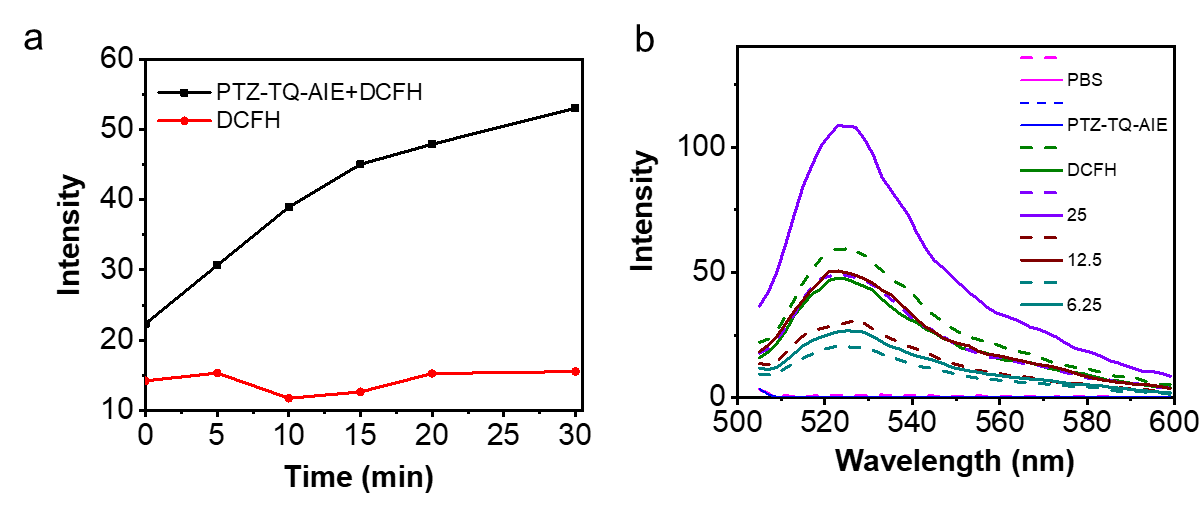


F**igure S18.** (a) ROS generation of PTZ-TQ-AIE dots with different concentrations. (b) ROS generation of PTZ-TQ-AIE dots with different times. The light source: 808 nm NIR laser (0.25 W cm^-2^) (unreal thread have no laser and real thread have laser).


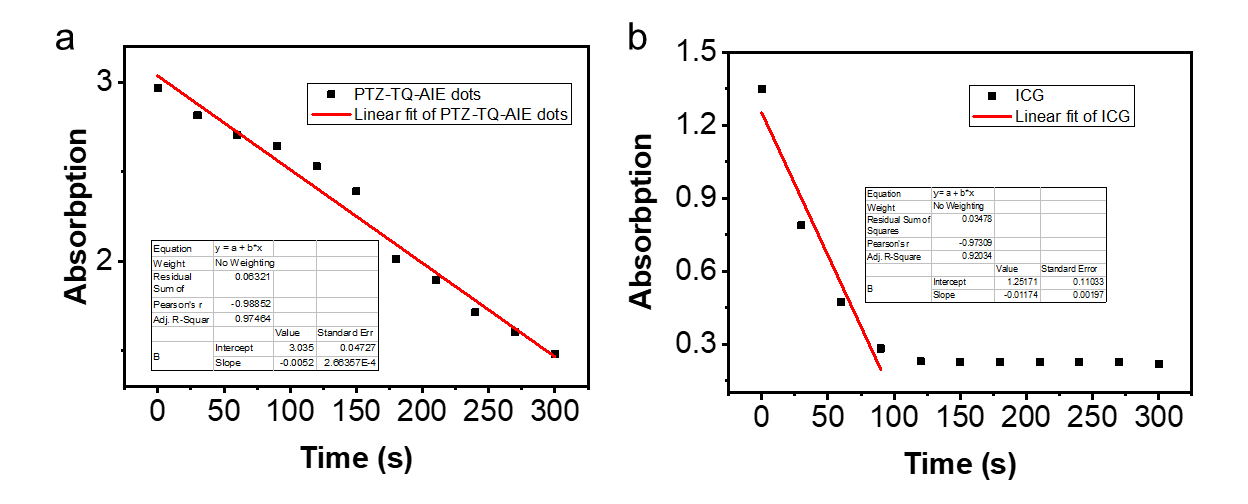


**Figure S19**. Decrease in absorbance of DPBF at 417 nm in the presence of PTZ-TQ-AIE dots (a) and ICG (b) as a function of irradiation time.


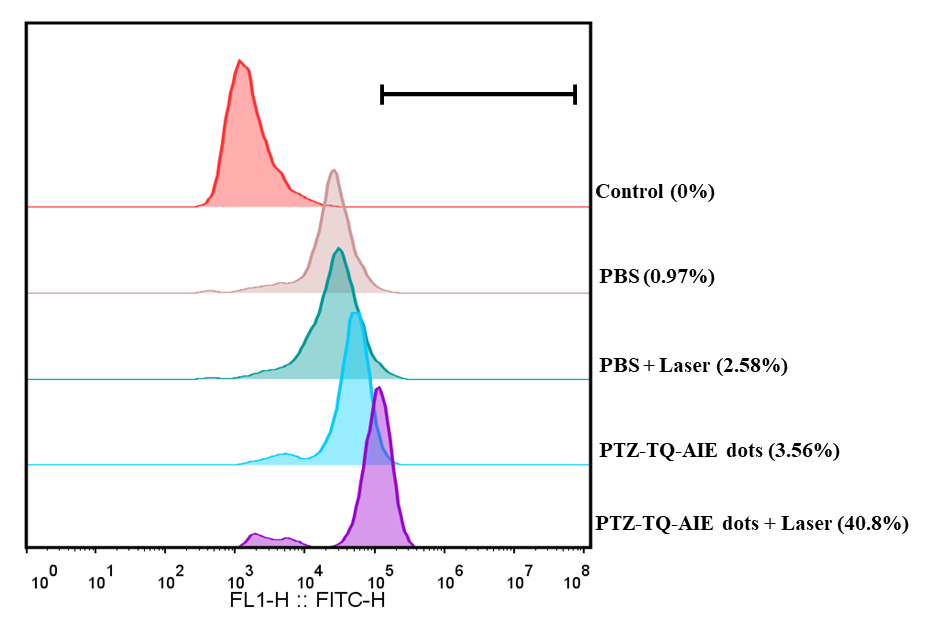


**Figure S20**. Singlet oxygen generation levels in HepG2 cells analyzed by flow cytometry.


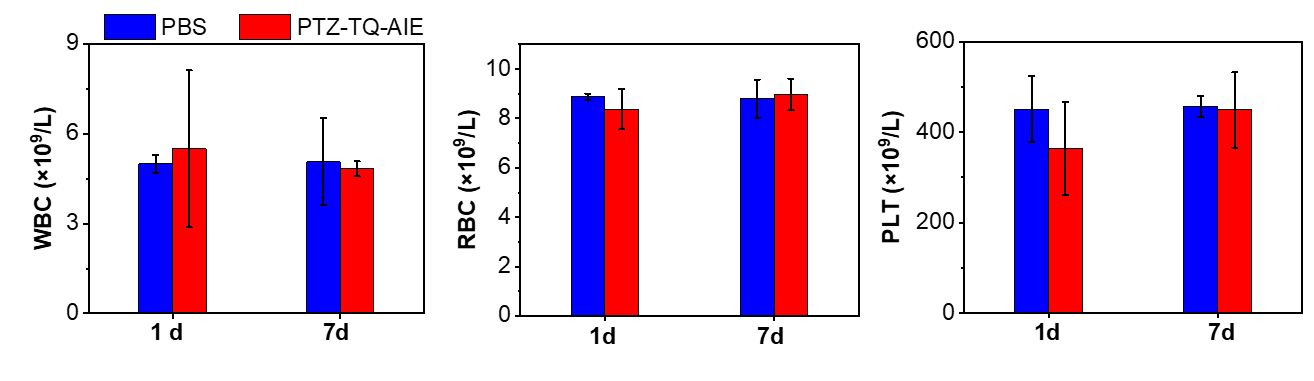


**Figure S21.** In vivo blood test including red blood cells, platelet, and white blood cell count of healthy mice injected with saline, PTZ-TQ-AIE dots for 1d and 7d.


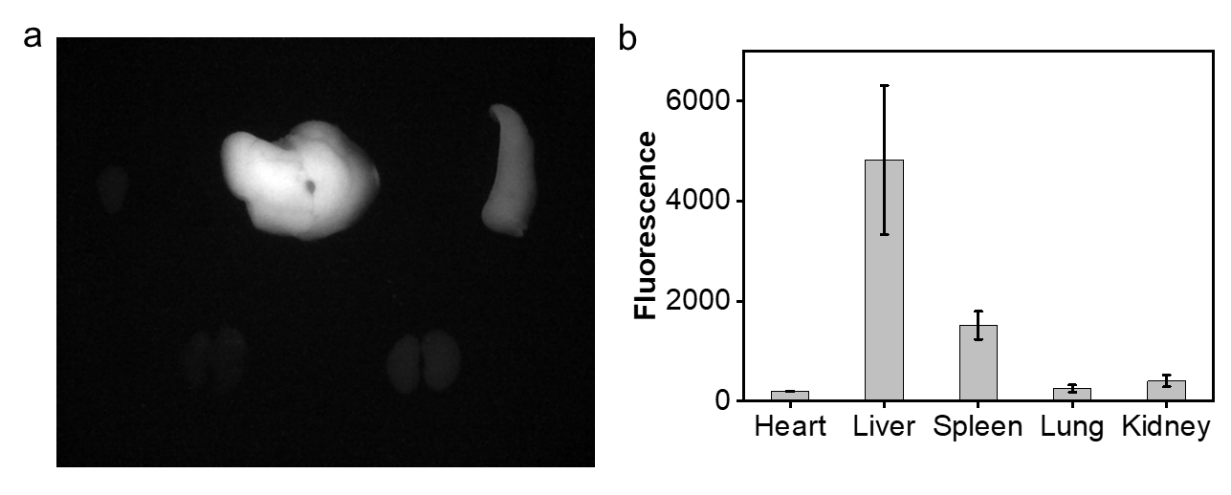


**Figure S22.** (a) The ex vivo biodistribution analysis of PTZ-TQ-AIE dots in BABL/c normal mice at 168 h under an 808 nm laser excitation (1250 nm bandpass filter, 300 ms). (b) The ex vivo fluorescent signal of different organs.

**Figure S23.** Blood circulation half-life curve of PTZ-TQ-AIE dots in mice. The circulation half-life was determined to be 61 minutes by fitting the data (5 min, 10 min, 20 min, 0.5 h, 1 h, 3 h, 6 h, 9 h, 12 h, 24 h, 30 h) to a first-order exponential decay (n = 3).


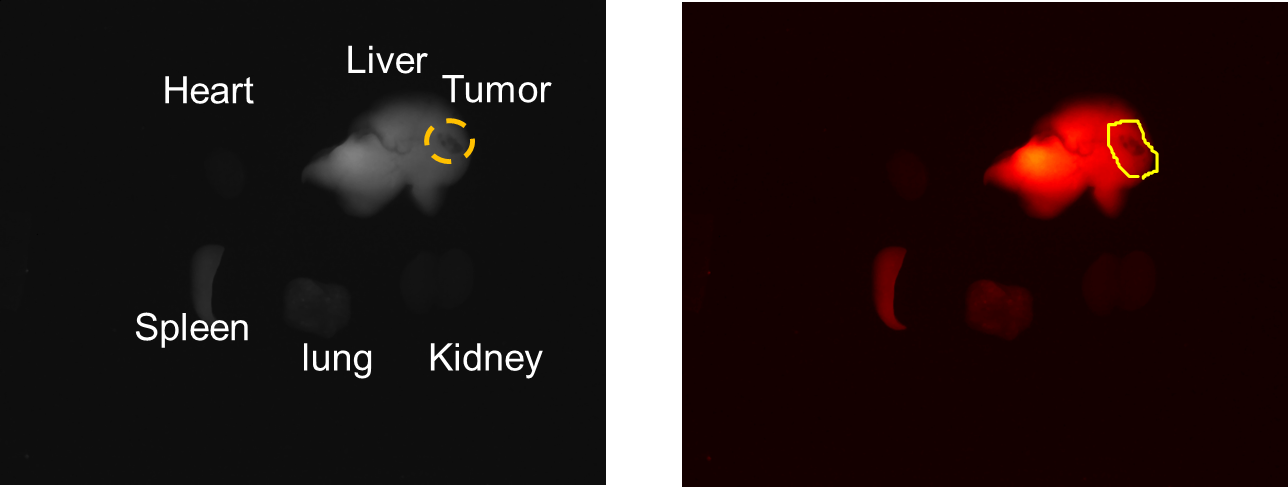


**Figure S24.** *Ex vivo* NIR-II fluorescent images of main organs collected after 2 h post injection.

**Figure S25.** The quantitative analysis of fluorescence intensity of orthotropic liver tumor at different time points after tail vein injection of PTZ-TQ-AIE dots (0.2 mL, 0.5 mg/mL).


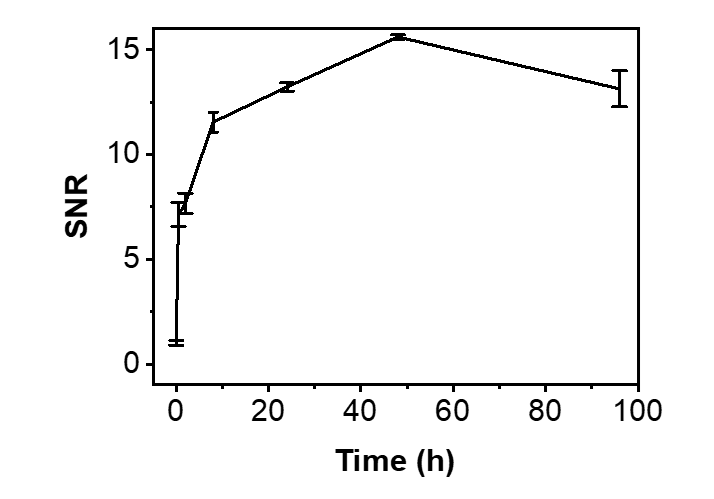


**Figure S26**. Signal-to-noise ratio (SNR) was analyzed in Figure 4c (n = 3).


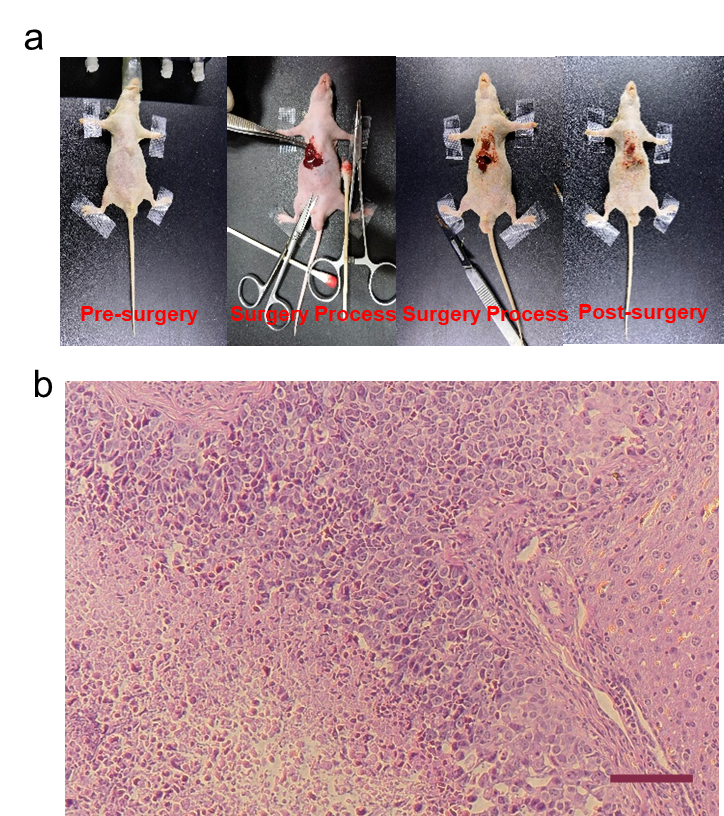


**Figure S27.** (a) Pictures of tumor resection process. (b) H&E staining of excised tumor and normal liver tissue (scale bar: 200 μm).


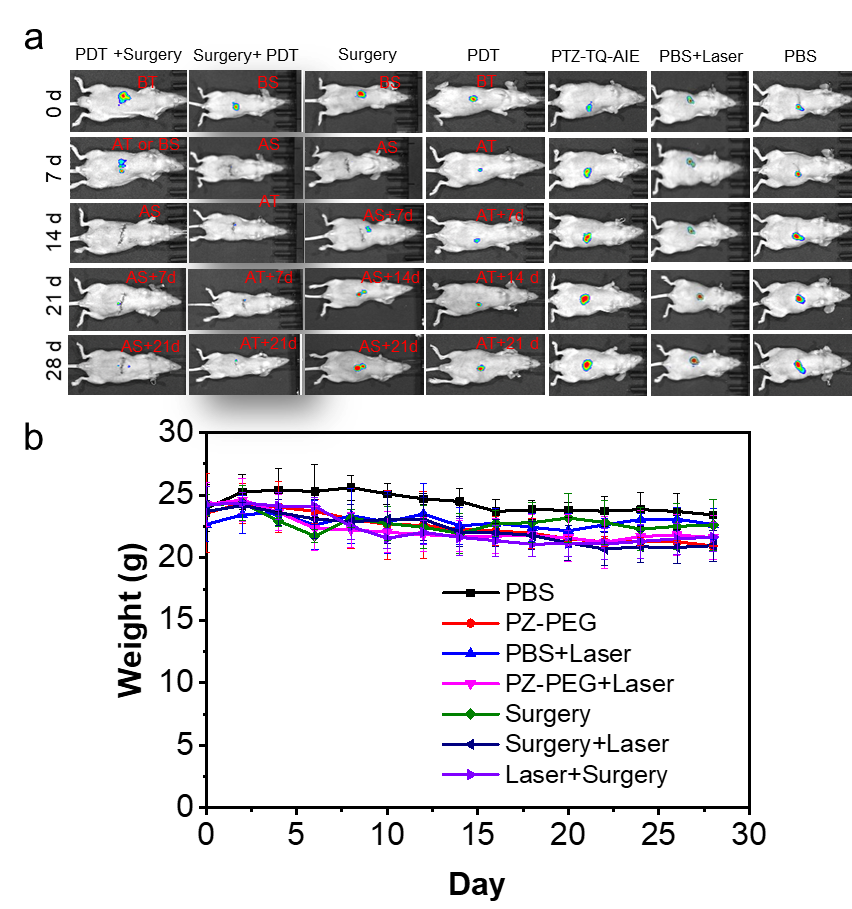


**Figure S28.** (a) Bioluminescence imaging of orthotopic liver cancer mice treated with PBS, PBS+Laser, PTZ-TQ-AIE dots, PDT, Surgery, Surgery+PDT, PDT+Surgery (BT: Before PDT; AT: after PDT; BS: Before surgery; AS: after surgery). (b) body weight curves after different treatments (n = 3).


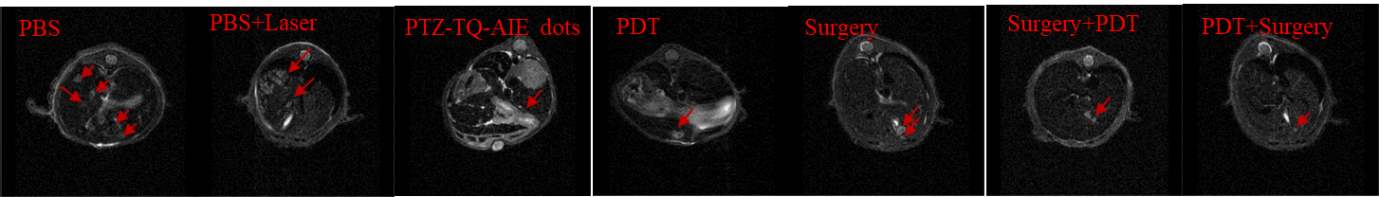


**Figure S29.** MR imaging of orthotopic liver cancer mice treated with PBS, PBS+Laser, PTZ-TQ-AIE dots, PDT, Surgery, Surgery+PDT, PDT+Surgery after therapy.


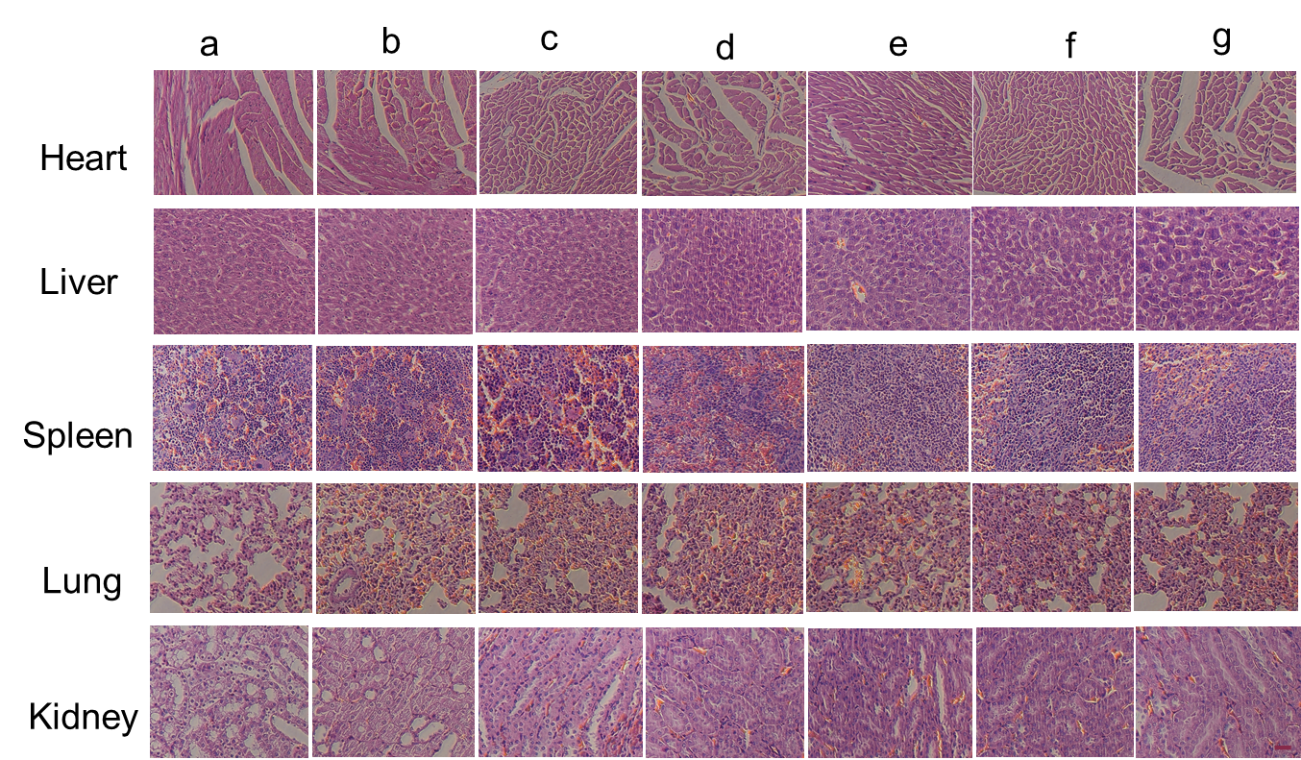


**Figure S30.** H&E staining of heart, liver, spleen, lung, and kidney tissue slices for different groups after treatments: (a) PBS, (b) PBS+Laser, (c) PTZ-TQ-AIE dots, (d) PDT, (e) Surgery, (f) Surgery+PDT, (g) PDT+Surgery.
