# Supplementary material for: NIR-II emissive AIEgen photosensitizers enable ultrasensitive imaging-guided surgery and phototherapy to fully inhibit orthotopic hepatic tumors
Source: J Nanobiotechnology. 2021 Dec 13;19:419. doi: 10.1186/s12951-021-01168-w (PMC8670198; doi:10.1186/s12951-021-01168-w)
Supplement: Supplementary file 2 — Additional file 2. Video of the NIR-II imaging guided surgery in orthotopic liver cancer model after tail vein injection of the PTZ-TQ-AIE dots 2 h and 48 h. [file 12951_2021_1168_MOESM2_ESM.pptx]

## Slide 1
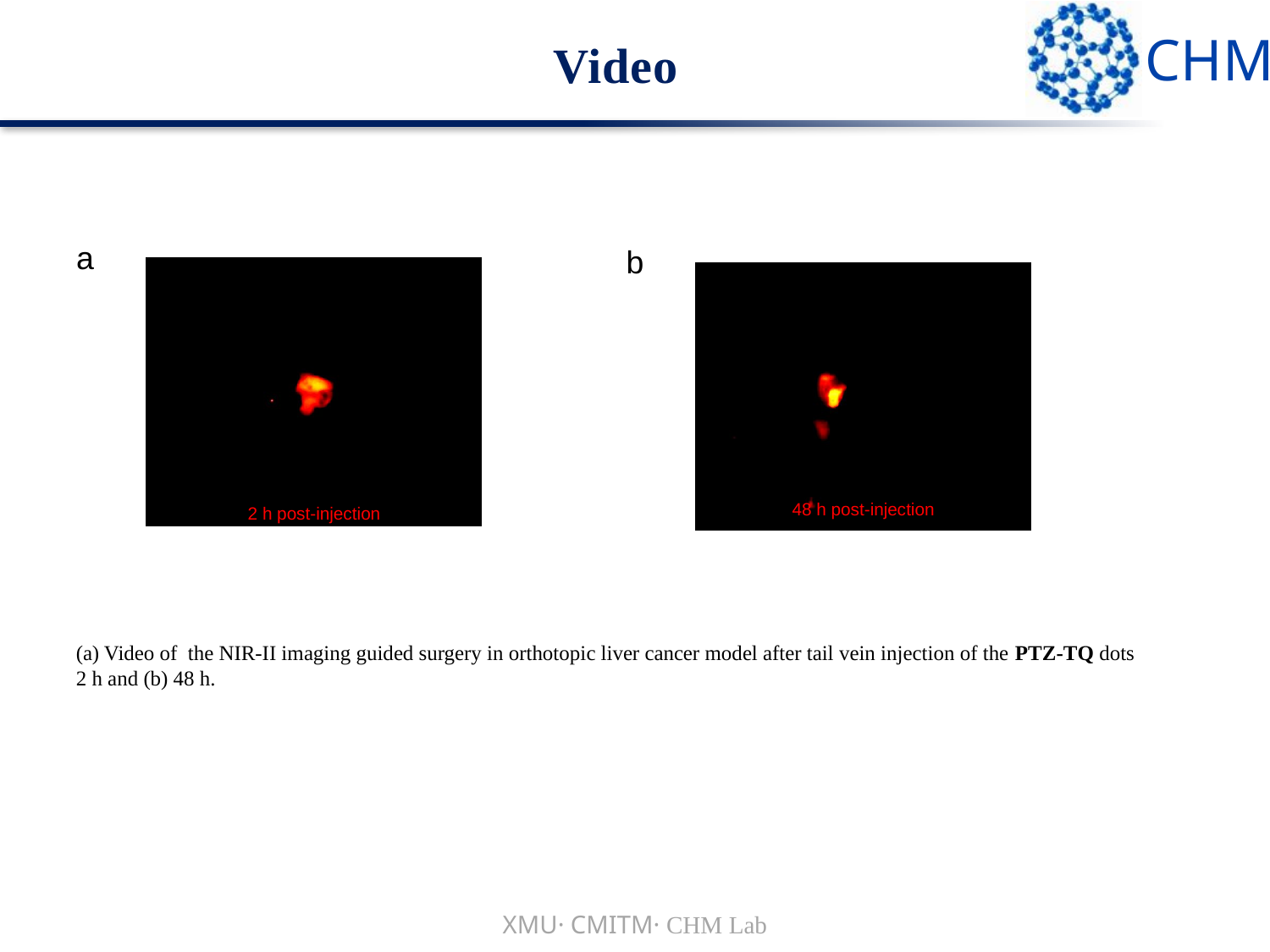

# Video
a
b
48 h post-injection
2 h post-injection
(a) Video of the NIR-II imaging guided surgery in orthotopic liver cancer model after tail vein injection of the PTZ-TQ dots 2 h and (b) 48 h.
